# Circulating *Helicobacter pylori* extracellular vesicles in serum as biomarkers of infection

**DOI:** 10.3389/fcimb.2026.1893888

**Published:** 2026-09-16

**Authors:** María Fernanda González, Gabriel Díaz-Serrano, Manuel Valenzuela-Valderrama, Marcelo J. Kogan, Alejandro Corvalán, Andrew F.G. Quest, Lisette Leyton

**Affiliations:** 1 Laboratory of Cellular Communication, Núcleo Interdisciplinario de Biología y Genética, Center for Studies on Extracellular vesicles, Metabolism and Cancer (CEMC), Institute of Biomedical Sciences (ICBM). Facultad de Medicina, Universidad de Chile, Santiago, Chile; 2 Advanced Center for Chronic Diseases (ACCDiS), Facultad de Ciencias Químicas y Farmacéuticas, Universidad de Chile, Santiago, Chile; 3 Faculty of Health Sciences, University of Talca, Talca, Chile; 4 Laboratorio de Carcinogénesis Molecular, Instituto de Investigación y Doctorados, Facultad de Medicina y Ciencias de la Salud, Universidad Central de Chile, Santiago, Chile; 5 Departamento de Química Farmacológica y Toxicológica, Facultad de Ciencias Químicas y Farmacéuticas, Universidad de Chile, Santiago, Chile; 6 Departamento de Hematología y Oncología, Facultad de Medicina, P. Universidad Católica de Chile, Santiago, Chile

**Keywords:** bacterial extracellular vesicles, diagnosis, *H. pylori* infection, inflammation, UreB

## Abstract

Gastric infection by *Helicobacter pylori* (*H. pylori*) is associated with extra-gastric pathologies. *H. pylori* can promote these pathologies by generating extracellular vesicles (Hp-EVs), which cross biological barriers and access other tissues through the circulation. However, the presence of Hp-EVs in the human circulation has not been demonstrated. We isolated and characterized EVs from serum of non-infected and infected patients, and used immunoblots (IB) to identify markers from host cells EVs (h-EVs) and Hp-EVs. We also assessed the predictive capacity of IB with an anti-Urease subunit B (UreB) antibody to identify true positive cases of *H. pylori* infection. Finally, we compared the diagnostic utility of IB-UreB with other detection methods of *H. pylori* in blood samples. Thirty symptomatic individuals underwent endoscopy, gastric biopsy and blood tests. Rapid Urease test (RUT), pathological analysis and *Gastropanel*® (GP) classified 16 cases (53.3%) as *H. pylori* positive. EV markers CD63 and CD81 were detected in all the isolated EVs from the serum of these patients. Moreover, UreB+ EVs were detected in 62.5% of *H. pylori*-infected patients, providing the first evidence of circulating Hp-EVs in human serum. Among the blood biomarkers evaluated, IB-UreB+ proved to be the best method for diagnosing *H. pylori*-positive cases. Furthermore, combining IB-UreB+ with GP increased its diagnostic value. Our findings provide the first evidence of circulating Hp-EVs in human serum, thereby supporting a mechanism for systemic dissemination of bacterial virulence factors. Additionally, their detection in blood samples may enable minimally invasive diagnosis of *H. pylori* infection.

## 1 Introduction

*H. pylori* is one of the most widespread human-infecting bacteria worldwide (FitzGerald and Smith, 2021). *H. pylori* is a spiral-shaped, microaerophilic, gram-negative bacterium that colonizes the human stomach (Marshall and Warren, 1984). While most individuals with this infection remain asymptomatic, in certain cases, it may contribute to the onset of diverse gastric pathologies, including gastritis, peptic ulcer, mucosa-associated lymphoid tissue lymphoma (MALT), and gastric adenocarcinoma (Alipour, 2021; FitzGerald and Smith, 2021). Additionally, infection with *H. pylori* is also associated with extra-gastric illnesses, such as hematological, dermatological, ophthalmic, and neurodegenerative diseases (Bravo et al., 2018; Gravina et al., 2020).

Multiple factors, including the immune system of the host and the presence of virulence factors (FitzGerald and Smith, 2021) in the *H. pylori* strain, influence the infection’s outcome. The most relevant and studied virulence factors are the proteins Cytotoxin-associated gene A (CagA), Vacuolating cytotoxin A (VacA), gamma-glutamyl transferase (GGT), and Urease, among others (Ansari and Yamaoka, 2019; de Brito et al., 2019; Valenzuela-Valderrama et al., 2019; Alipour, 2021). Moreover, *H. pylori* is capable of releasing extracellular vesicles, hereafter referred to as Hp-EVs (Ansari and Yamaoka, 2019). Hp-EVs are generated when protrusions on the bacterial membrane detach from the surface, capturing components of the periplasm and membrane proteins (Heczko et al., 2000; González et al., 2021). The size of Hp-EVs varies from 20 to 450 nm (Palacios et al., 2023) and, due to their small size, they are thought to cross several biological barriers, being able to pass from the stomach into the bloodstream and from there to other organs and tissues. Hp-EVs also include bacterial virulence factors, so they can mediate detrimental effects on recipient cells, and thus Hp-EVs themselves are also considered virulence factors. Of note, a few studies have reported the presence of EVs originating from pathogenic bacteria within the human circulatory system (Tulkens et al., 2020; González et al., 2021).

On the other hand, EVs derived from host cells (h-EVs) represent a form of intercellular communication, both locally and remotely, and exhibit substantial prevalence in the circulatory milieu (Iannotta et al., 2024). Therefore, the scarcity of studies identifying bacterial EVs in the human circulatory system is likely due to the difficult challenge of differentiating pathogen-derived EVs from h-EVs. Indeed, the size range, a key feature frequently employed to purify EVs, for h-EVs typically falls within 50 to 1000 nm (Campos et al., 2019), exhibiting a considerable overlap in size with the bacterial EV populations. Furthermore, h-EVs are more abundant in the bloodstream than bacterial EVs generated during bacterial infection (An et al., 2018; Tulkens et al., 2020), making their detection in the circulation particularly challenging.

Our recent findings demonstrate that Hp-EVs induce astrogliosis and neuronal damage both *in vitro* and *in vivo*, in a murine model (Palacios et al., 2023). In that study, intravenous and intragastrical administration of Hp-EVs via oral gavage resulted in their appearance in the brain of the mice. Furthermore, comprehensive proteomic profiling revealed that Urease was one of the most abundant proteins in Hp-EVs, which was also detected in the brain of Hp-EV-treated mice. However, the results shown by Palacios et al. (2023), were based on a pre-clinical model. Therefore, there is still no experimental evidence that Hp-EVs can cross the epithelial/endothelial barriers of the stomach and reach the bloodstream of infected human subjects.

*H. pylori* Urease has long been recognized as a survival factor; however, increasing evidence from our group and others supports an additional role for this protein in cell signaling (Fan et al., 2000; Beswick et al., 2006; Palacios et al., 2023). *H. pylori* Urease is a nickel-dependent metalloenzyme that plays a central role in gastric colonization and bacterial survival in the stomach’s acidic environment (van Vliet et al., 2001). It catalyzes the hydrolysis of urea to generate ammonia and bicarbonate, which helps neutralize the pH of the periplasmic and cytoplasmic microenvironment (Mobley et al., 1995). Structurally, native Urease is composed of two subunits —A (UreA) and B (UreB)— organized into a high-molecular-weight multimeric complex consisting of six copies of each, with active sites containing two nickel ions essential for catalysis (Cunha et al., 2021).

In the present study, we sought to detect the systemic distribution of Hp-EVs using immunoblots (IB) to detect *H. pylori* Urease in isolated EVs from serum of patients infected with the bacteria, since *H. pylori* Urease is an abundant and specific biomarker of Hp-EVs (Palacios et al., 2023).

Given that Hp-EVs function as vehicles for virulence factors, their presence in the circulation implies that they could disseminate from the circulatory system to other organs. This fact could help elucidate the association of *H. pylori* infection with extra-gastric diseases. Furthermore, the presence of Hp-EVs in the circulatory system holds potential significance as a biomarker, offering an alternative method to diagnose *H. pylori* and aligning with the concept of liquid biopsy through the detection of biomarkers in EVs from blood (Möhrmann et al., 2018).

## 2 Materials and methods

### 2.1 Purification of *H. pylori* Extracellular Vesicles (Hp-EVs)

To determine the detection limits of CagA, UreB, and VacA, purified Hp-EVs from strain 60190 were analyzed by immunoblot. For this, the *H. pylori* strain 60190 (ATCC 49503) was grown in liquid culture using Brucella broth with 0.3% β-cyclodextrin (Sigma-Aldrich, Burlington, Massachusetts, USA), *H. pylori* selective supplement Dent (Oxoid, Basingstoke, Hampshire, England), and Vitox (Oxoid, Basingstoke, Hampshire, England), under microaerophilic conditions at 37 °C for 72 h. Hp-EVs were purified following the protocol described by Palacios, E. et al., 2023. Briefly, 250 ml of *H. pylori* culture was centrifuged at 2,000 x *g* for 10 min at 4 °C. The supernatant was then filtered through 0.2 µm filters and concentrated to ~1 ml using Amicon® Ultra-15 Centrifugal Filters (100 kDa MWCO Amicon, Millipore, Merck). Later, one volume of concentrated media was mixed with 0.5 volume of the Exo-spin precipitation buffer #EX-01 (Cell Guidance System LLC, Helix Center, USA) overnight at 4 °C and then centrifuged at 16,000 x *g* and 4 °C for 1 h. The pellet was resuspended in 100 µl of phosphate-buffered saline (PBS, filtered with 0.1 µm filters, Millipore, Merck) and purified using an exclusion chromatography column (Exosome Spin Column MW3000, Invitrogen, Carlsbad, California, USA).

### 2.2 Patients

Thirty patients were included in this retrospective study, comprising both females and males. The patients were enrolled from the Hospital San Juan de Dios de Curico, Servicio de Salud del Maule and Hospital Puerto Montt Dr. Eduardo Schutz Schroeder, Servicio de Salud de Los Lagos, Chile. All patients provided written informed consent. Patients presented to the hospitals with a variety of gastrointestinal symptoms and subsequently underwent upper endoscopy with gastric biopsy, during which, a Rapid Urease Test (RUT) was performed. Both endoscopic and histopathological reports were obtained from these procedures. Additionally, demographic data—including sex and age—were recorded, and a peripheral blood sample was collected from each patient. Serum was extracted from these blood samples and stored at -80 °C for approximately 5 years for subsequent analyses. All clinical and demographic data was de-identified for the purpose of this research. To standardize the EV purification protocol, serum samples were also collected from healthy volunteers. Blood was drawn following standard venous sampling procedures, centrifuged at 3,000 x *g* for 10 min at 4 °C, and the serum was transferred to sterile tubes and stored at -80 °C until further use. All procedures involving human subjects were conducted according to the institutional guidelines and approved by the Ethics Committee of the Pontificia Universidad Católica de Chile and University of Talca (Number 2016-03-NB) under ANID-FONDECYT project number 1151411.

### 2.3 Purification of extracellular vesicles from serum

The initial serum (1–2 ml) was pre-processed as follows: 1) a dilution of the sample to 6 ml with PBS to decrease sample viscosity, 2) a centrifugation at 2,000 x *g* for 10 min and, 3) a filtration of the supernatants using 0.22 µm filters, to remove cell debris and large EVs. For protocol *(a)* UC-qEV, the filtered supernatant was ultracentrifuged (100,000 x *g*) for 90 min, the pellet was resuspended in 500 µl of PBS and loaded onto qEV original (70 nm series) columns from IZON^®^ erase (Christchurch, New Zealand), and fractions 1, 2 and 3 were collected following the manufacturer’s instructions. Briefly, the sample was loaded onto the column and allowed to enter completely before adding PBS (filtered with 0.1 µm filters, Millipore, Merck) to the column. Three fractions of 500 µl each were collected after discarding the void volume of the column. For protocol *(b)* qEV, the filtered supernatant was concentrated to 500 µl using Amicon^®^ (Ultra-15 Centrifugal Filter, 100 kDa MWCO Amicon, Millipore, Merck), loaded onto qEV columns and then fractions 1, 2 and 3 were collected as previously mentioned. For protocol *(c)* qEV2, the protocol *(b)* was followed, and the three fractions collected were concentrated to 500 µl using Amicon^®^, and loaded again into qEV columns, to collect fractions 1, 2 and 3. The optimized protocol consisted of an adapted version of protocol *(b)* the qEV, which included several additional steps to enhance yield and minimize sample loss. Specifically, the three fractions were concentrated to ~70 µl using Amicon^®^ Ultra Centrifugal Filters (30 kDa MWCO) pre-incubated with 0.4% polyvinylpyrrolidone (PVP-40, Sigma-Aldrich, Burlington, Massachusetts, USA) to prevent non-specific adsorption of EVs to the membrane. The resulting purified EVs from each fraction were then characterized by NTA to determine their size and concentration, total protein concentration, and IB to evaluate the presence of canonical EV markers (CD63 and CD81) and UreB, used as a marker of *H. pylori* infection.

### 2.4 Nanoparticle tracking analysis (NTA)

The determination of EV size and concentration was performed by NTA (Bendre et al., 2011). This analysis uses video recordings of Brownian motion to estimate the size. Samples were diluted 1:100 in PBS (filtered with 0.1 µm filters, Millipore, Merck) to obtain 10 to 100 particles per frame. This final suspension was injected into the Nano Sight NS300 (Malvern Panalytical). For each sample, three videos of 30 s were recorded, which were then analyzed using the Nano Sight NTA 3.2 software to estimate the vesicle size and concentration.

### 2.5 Scanning electron microscopy (SEM)

The EVs were characterized by SEM. After fixing the samples with 1% glutaraldehyde for 5 min, they were deposited on FCF300-Cu grids (Sigma-Aldrich) and dried with absorbent paper. The grids were then washed with water. Then, 15 µl of phosphotungstic acid (0.1%) was added and incubated for 30 s before absorbing the excess liquid with filter paper. Finally, the grids were washed again with water. Images were acquired with an INSPECT-F50 high-resolution SEM (Thermo Fisher Scientific, FEI, The Netherlands), with a STEM detector.

### 2.6 Total protein concentration and immunoblot analysis

Total protein extracts from the EVs were obtained by sonication in a buffer containing 20 mM HEPES (pH 7.4) (Gibco, Grand Island, New York, USA), 0.1% SDS (USBiological Life Sciences, Swampscott, Massachusetts, USA), 0.05% NP-40 (Sigma-Aldrich, Burlington, Massachusetts, USA), and a cocktail of protease inhibitors: 12.5 µg/ml leupeptin (Calbiochem, San Diego, California, USA), 10 µg/ml antipain (Calbiochem, San Diego, California, USA), 100 µg/ml benzamidine (Calbiochem, San Diego, California, USA), 1 mM phenylmethylsulphonyl fluoride (Sigma-Aldrich, Burlington, Massachusetts, USA), 1 mM sodium orthovanadate (Sigma-Aldrich, Burlington, Massachusetts, USA), and 10 mM sodium fluoride (Sigma-Aldrich, Burlington, Massachusetts, USA). Total protein concentration was determined using the Pierce BCA Protein Assay Kit (Thermo Fisher Scientific, Waltham, Massachusetts, USA) with BSA Protein Standards. Total proteins (3–10 μg per lane) were separated by SDS-PAGE on a 10% polyacrylamide gel and transferred onto nitrocellulose membranes Amersham™ (Cytiva, Marlborough, Massachusetts, USA). Membranes were blocked with PBS containing 0.1% Tween and 5% skim milk for 1 h. The membranes were incubated overnight at 4 °C with primary antibodies diluted in the same blocking buffer, under gentle agitation: anti-CD63 (1:3,000), anti-UreB (1:5,000) both from Abcam, (Cambridge, MA, USA, # ab68418 and #ab127916 respectively); anti-CD81 (1:1,000), anti-CagA (1:1,000), and anti-VacA (1:1,000) all from Santa Cruz Biotechnology (Dallas, TX, USA, #sc-166029, #sc-28368, #sc-32746, respectively). Primary antibodies were detected with HRP-conjugated secondary antibodies and the EZ-ECL kit (Biological Industries, Beit Haemek, Israel).

### 2.7 Statistical analysis

To compare two groups, we employed the unpaired t-test. A two-way ANOVA was used to compare three or more groups, followed by Tukey’s multiple comparisons test. Data are expressed as the mean ± standard error of the mean (S.E.M.) in all cases. To create a predictive model, we used univariable and multivariable logistic regression with data from thirty patients (n=30). The odds ratio (OR) and 95% confidence intervals (95% CI) were calculated using the profile likelihood method. In all models, the Urease test results from gastric biopsies served as the dependent variable. For univariable models, the independent variables included: immunodetection of *H. pylori* Urease from EVs (IB-UreB+), gastrin-17, serum anti-Hp antibodies, serum pepsinogen I/II ratio (PGI/PGII), and the *GastroPanel* (GP) report. The multivariable model was constructed using IB-UreB+ in combination with the GastroPanel (GP) report. Model selection was determined by the corrected Akaike Information Criteria (AICc) difference relative to the intercept-only model and the log-likelihood test (G-square). For each model, we constructed Receiver Operating Characteristic (ROC) curves to compute the Area Under the Curve (AUC), sensitivity, specificity, negative predictive value (NPV), positive predictive value (PPV), and accuracy. Statistical significance was defined as P ≤ 0.05, while P > 0.05 was considered non-significant (ns). All analyses were conducted using GraphPad Prism V9.

## 3 Results

### 3.1 Discovering a biomarker to detect *H. pylori* Extracellular Vesicles in human serum

Since our goal was to detect Hp-EVs in the human bloodstream, a comprehensive literature search was conducted to identify their most prevalent proteins. Although the analysis of Hp-EVs by proteomic profiling remains limited (Zavan et al., 2019; Palacios et al., 2023; Johnston et al., 2024), we found that the virulence factors CagA, UreB, and VacA were plausible candidates for the identification of Hp-EVs using immunoblot (IB) assays. Consequently, to evaluate the most effective virulence factor indicative of Hp-EVs presence in blood circulation, decreasing amounts of purified Hp-EVs from the strain 60190 were loaded on a gel and subjected to IB to determine the detection limit of CagA, UreB, and VacA. To this end, Hp-EVs were purified from liquid cultures using a previously reported methodology (Palacios et al., 2023), summarized in the Methods section. After quantifying the total protein concentration of the EV preparations, CagA, UreB, and VacA were monitored by IB using specific antibodies (**Figure 1**). CagA was detected in Hp-EVs at concentrations as low as 150 ng. UreB was successfully identified at even lower protein concentrations (50 ng). Finally, the detection of VacA was the least successful marker, being detected only when 1500 ng of Hp-EVs were analyzed, despite the use of long exposure times (3 min). In accordance with our previous proteomic analysis, which identified Urease (UreA and UreB) as being amongst the most abundant proteins in these Hp-EVs (Palacios et al., 2023), the present findings support considering UreB as the best marker among those tested to detect Hp-EVs. We therefore propose UreB+ vesicles as a candidate biomarker associated with current *H. pylori*-positive status in serum.

**Figure 1:**
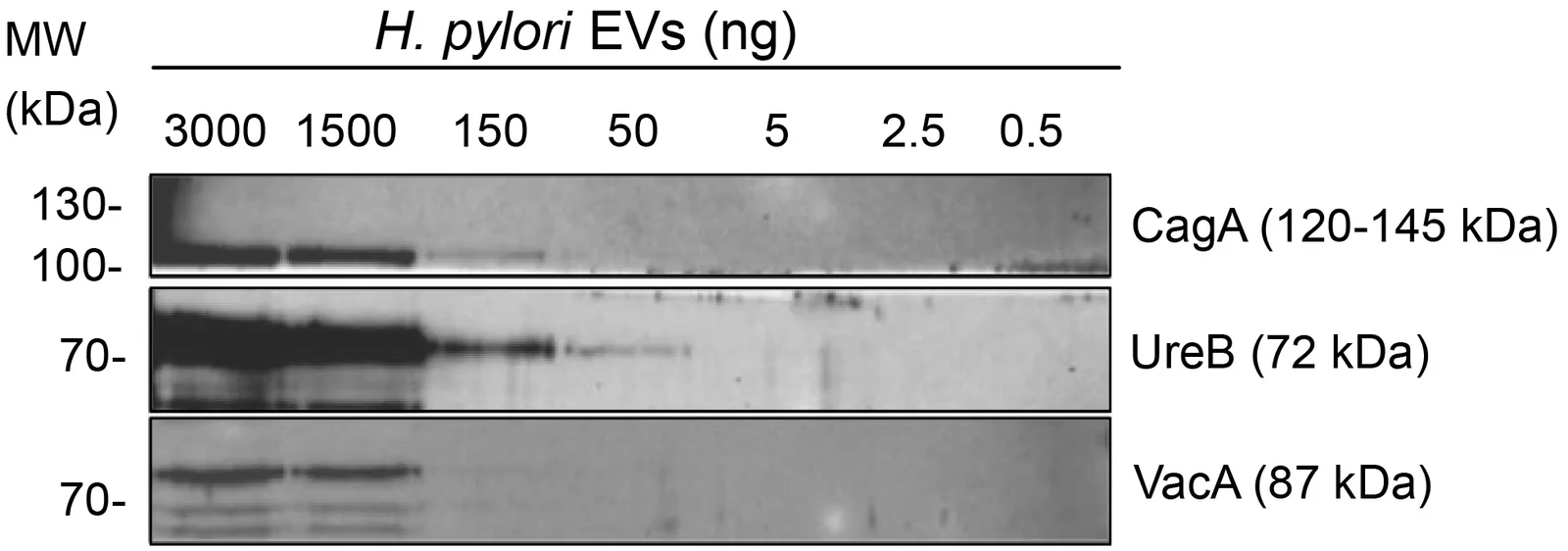
Detection levels of CagA, UreB, and VacA in *H. pylori* Extracellular Vesicles (Hp-EVs). Hp-EVs were purified, quantified, and loaded on a gel using various protein quantities (3000 ng– 0,5 ng). Levels of CagA, UreB, and VacA were assessed by immunoblotting (IB). The detection was performed using the EZ-ECL kit*. Molecular weight (MW) values in kDa are indicated to the left. * Uncropped IBs are in Supplementary file.

### 3.2 Purification of extracellular vesicles from human serum

To assess the proposed hypothesis, we first purified the EVs from human serum. To this end, we tested three different methodologies using serum from healthy volunteers (**Figure 2**). The methodologies began with the preprocessing of the samples: first, we diluted the serum with PBS to reduce its viscosity, centrifuged at 2,000 x *g* for 10 min, and the supernatant was filtered with 0.2 µm filters. Then, three different protocols were carried out: *a)* UC-qEV: a combination of ultracentrifugation with size exclusion chromatography (qEV original IZON columns), *b)* qEV: size exclusion chromatography (qEV original IZON columns), and *c)* qEV^2^: two-fold purification through size exclusion chromatography (qEV original IZON columns) (**Figure 2**). Three fractions of each of these three protocols were collected (**Figure 2**) and analyzed using NTA. Their protein content was quantified by BCA Protein Assay Kit and analyzed by IB. Similar-sized vesicles were obtained with the three protocols (**Figure 3A**). However, EV concentration and total protein concentration varied among the methods and fractions, with the highest values obtained for fraction 3 using the qEV protocol (**Figures 3B, C**). Moreover, the EV marker CD81 was only detected in fraction 3 from the qEV protocol, whereas CD63 was detected in almost all fractions (**Figure 3D**). Since the qEV protocol (*b*), was the protocol that produced the best results, we selected this procedure to proceed further with our research. To optimize our chosen methodology, we performed additional steps. This improvement involved a concentration step using Amicon^®^ (Ultra Centrifugal Filter with a 30 kDa molecular weight cutoff) to concentrate the samples to ~70 µl (**Figure 4A**). To diminish sample loss, the filters were previously incubated with 0.4% PVP-40. Using the optimized protocol, we purified EVs from the sera of healthy volunteers, and we characterized them by NTA, quantification of total proteins, and IB. The mean size determined by NTA (**Figure 4B**), was similar in the three fractions obtained. The concentration of particles (particles/ml) in fraction 3 was significantly higher and slightly higher, compared to fractions 1 and 2, respectively (**Figure 4C**). Also, total protein concentration revealed that fraction 3 exhibited significantly higher and slightly higher protein concentrations than fraction 1 and fraction 2, respectively (**Figure 4D**). Next, we elaborated the profiles of the EVs (concentration vs. size) of the three fractions using the data obtained by the NTA analysis (**Figure 4E**), which evidenced that fractions 2 and 3 displayed a homogeneous profile; in addition, fraction 3 had a higher concentration of EVs compared to fraction 2. The *inset* in **Figure 4E** enlarges the lower part of the graph to clearly show the profile of fraction 1, which was more heterogeneous in size and had lower particle concentrations than the other fractions. Finally, IB of fractions 2 and 3, revealed the EV markers CD63 and CD81 (**Figure 4F**), which were more noticeable in fraction 3 than in fraction 2. The final protocol to isolate EVs from serum resulted in the collection of fraction 3 using the optimized protocol (**Figure 4A**). The resulting protocol represents an optimized and more efficient method to purify EVs, improving the overall performance of the experimental procedure.

**Figure 2:**
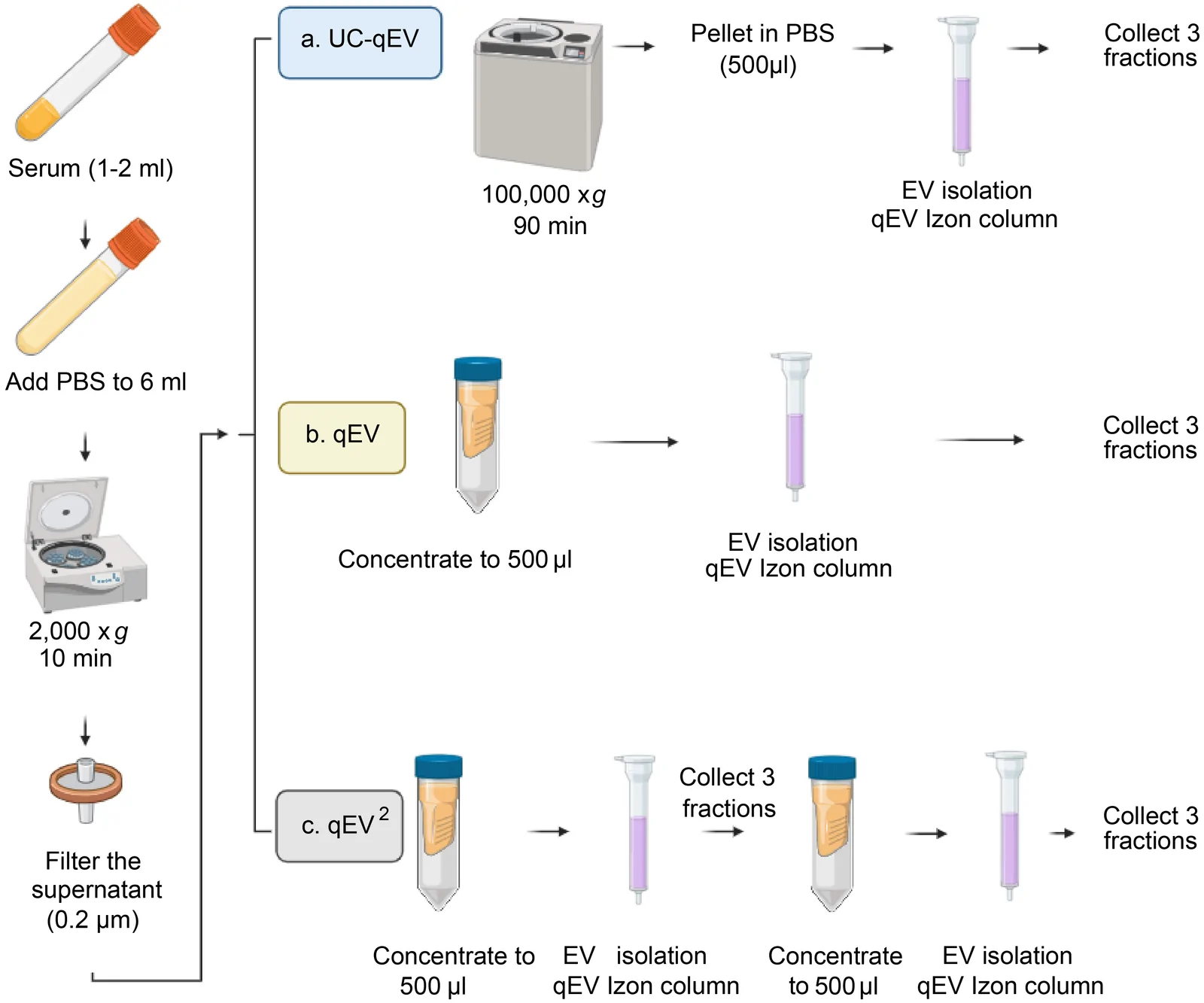
Three different methods for the purification of EVs from human serum. Scheme of the three protocols (for more details see the Materials and methods section). Three fractions were obtained and characterized from each protocol.

**Figure 3:**
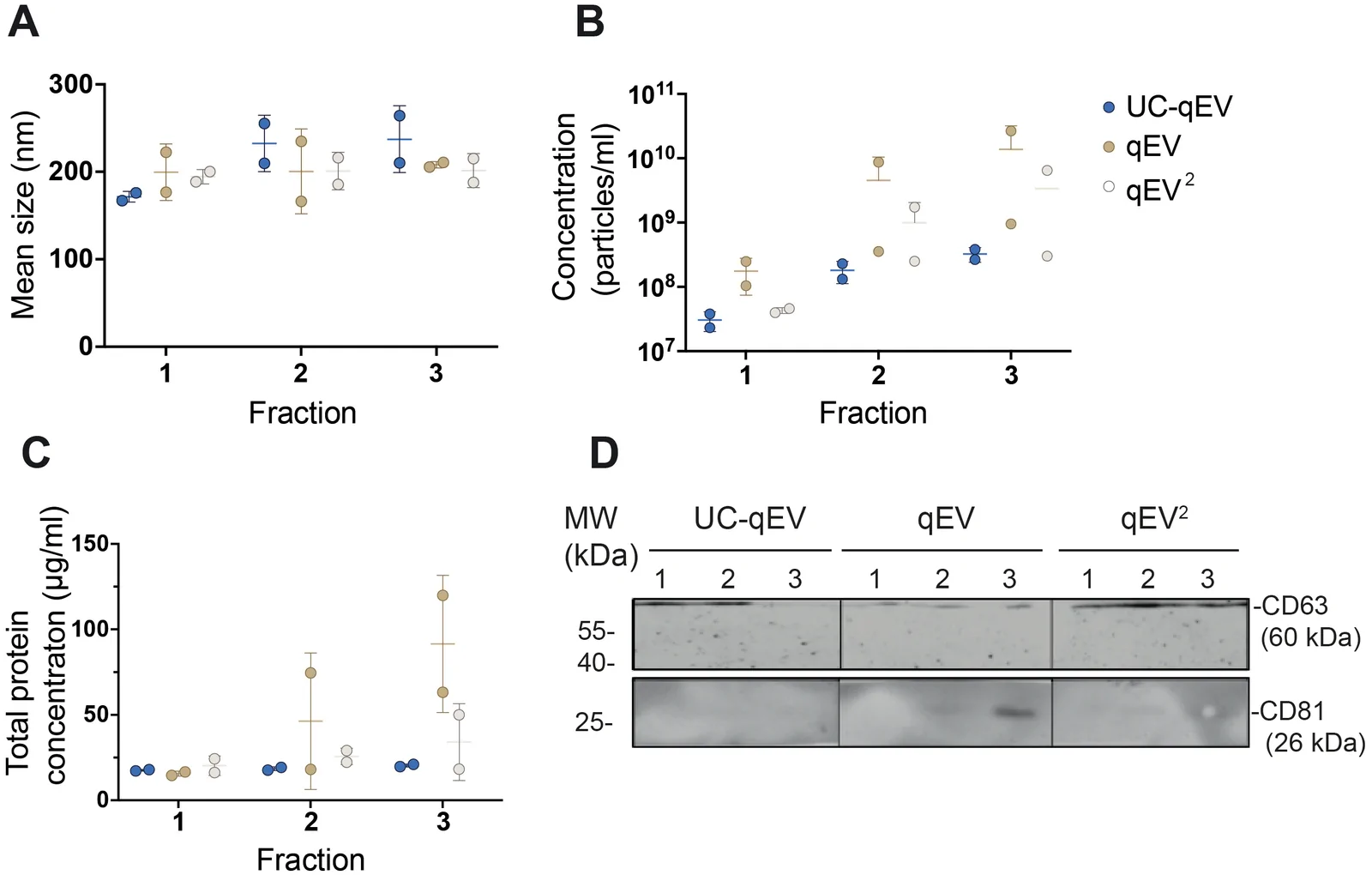
Evaluation of three different methods for the purification of EVs from human serum. The EVs obtained in the three fractions collected from each method were analyzed by nanotracking (NTA) to determine the mean size of the particles **(A)** and the concentration expressed in particles/ml **(B)**, quantification of total protein concentration using the BCA Protein Assay Kit **(C)**, and IB to evaluate the presence of the CD63 and CD81 EV markers **(D)**, Molecular weight (MW) values in kDa are indicated to the left*. In A, B, and C, each point represents the value of each fraction isolated from 2 independent experiments; the mean and SD are indicated. In D, the numbers 1, 2, and 3 represent each corresponding fraction. The EVs were obtained using serum samples from two volunteers. * Uncropped IBs are in Supplementary file.

**Figure 4:**
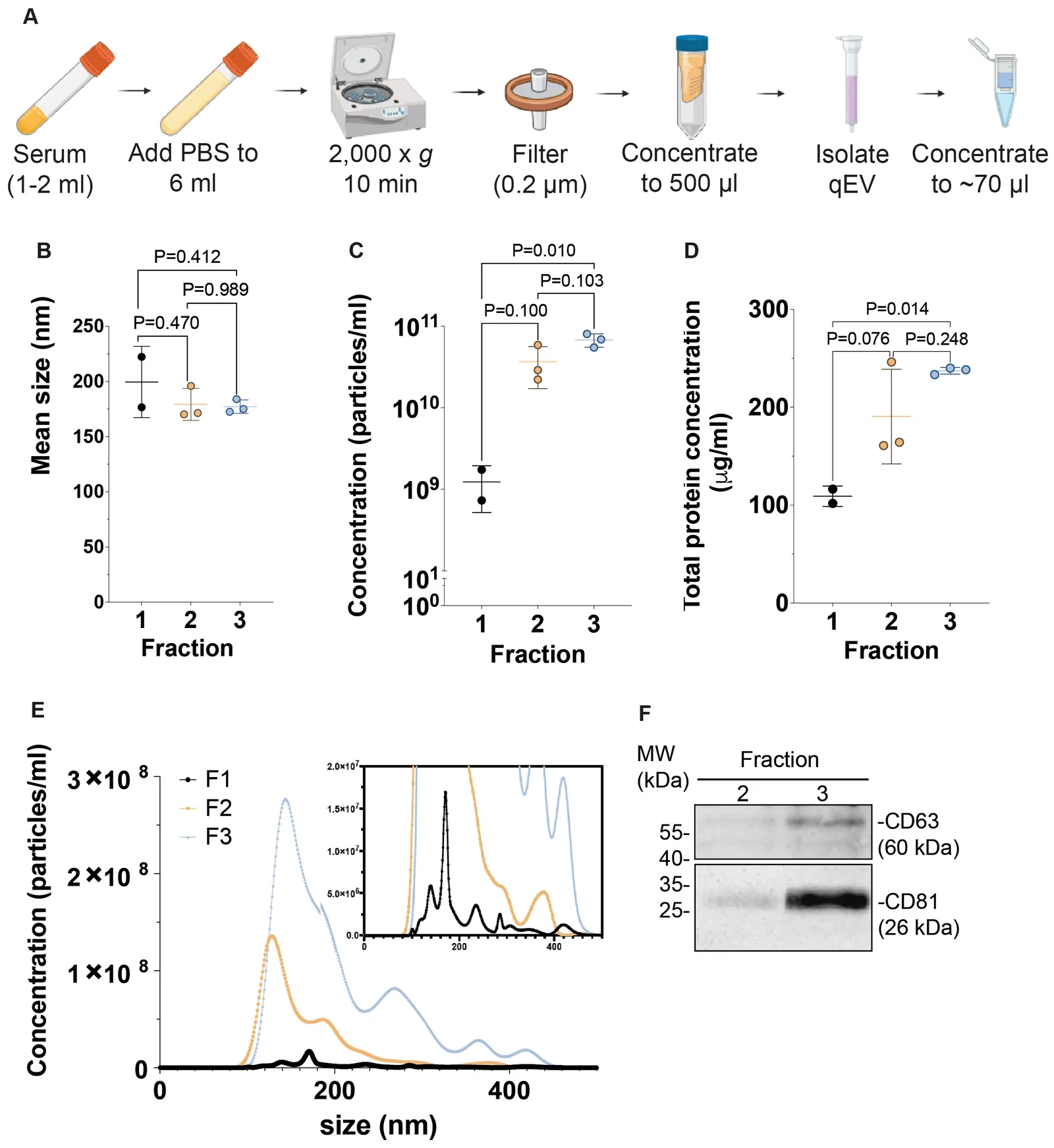
Optimized protocol for the purification of EVs from the serum of patients. The methodology developed was based on the optimization of the qEV method shown in **Figure 2**. **(A)** Samples were diluted, subjected to centrifugation, and filtration. Samples were then concentrated using Amicon^®^, and the EVs isolated using the qEV columns. Finally, fractions were concentrated using Amicon^®^. The EVs contained in the concentrated fractions 1, 2, and 3 were characterized by nanotracking (NTA) to determine the mean size **(B)** and the concentration **(C)** of the particles obtained. The total protein concentration **(D)** was determined using the BCA Protein Assay Kit. **(E)** EV profiles (concentration vs. size) were elaborated using the data obtained by the NTA. The inset is an enlargement of the lower part of the curves. **(F)** Immunoblot to detect the CD63 and CD81 EV markers*, Molecular weight (MW) values in kDa are indicated to the left. In B, C, and D, each point represents the value of a fraction isolated from three independent experiments, except for fraction 1 (n=2); the mean and SD are also represented. Statistical analyses were performed using ANOVA. Exact p values are reported in the figure. * Uncropped IBs are in Supplementary file.

### 3.3 Patient clinical data

Patients included in this study were referred for an upper endoscopy based on gastrointestinal symptoms. These patients also underwent a Sydney gastric biopsy protocol and RUT. A blood sample was also taken for GP analysis. Thirty patients were randomly selected; true positive cases (n = 16) were defined as those confirmed by both the positive RUT and histopathological report. Discordant cases were further resolved using the GP as a third reference test. Gastric cancer status and staging according to the Operative Link for Gastritis Assessment (OLGA) and (Operative Link on Gastric Intestinal Metaplasia Assessment (OLGIM) systems were reported. De-identified patient’s age and sex were also documented (**Table 1**). No significant age differences existed between the *Hp-* and *Hp+* groups (**Figure 5A**). The gender distribution of females and males was also similar between the two groups. (**Figure 5B**). The patients were recruited from the high risk of gastric cancer and high prevalence of *H. pylori* infection regions in Chile (**Figure 5C**) (https://diprece.minsal.cl/garantias-explicitas-en-salud-auge-o-ges/guias-de-practica-clinica/cancer-gastrico/descripcion-y-epidemiologia/).

**Table 1:** Clinical characteristics of the patients included in the study.

ID	Gastric Cancer	RUT	*H. pylori* in pathology report	Intestinal metaplasia in pathology report	OLGA	OLGIM	Age	Sex
NV-01	0	0	1	0	I	I	50	Male
NV-02	1	0	0	1	0	0	65	Female
NV-03	0	0	0	0	0	0	62	Male
NV-04	0	0	0	1	IV	I	56	Male
NV-05	0	0	1	1	II	0	69	Female
NV-06	0	1	1	0	0	0	46	Male
NV-07	0	1	1	0	0	0	65	Female
NV-08	0	1	1	0	I	0	64	Male
NV-09	0	1	1	0	I	I	54	Female
NV-10	1	1	1	1	I	0	64	Female
NV-11	0	0	1	1	I	I	48	Female
NV-12	0	0	0	0	III	0	40	Male
NV-13	0	1	1	0	I	0	57	Male
NV-14	0	1	1	0	0	0	62	Female
NV-15	0	1	0	0	0	0	56	Male
NV-16	0	1	0	0	II	0	47	Male
NV-17	0	1	1	0	0	0	50	Female
NV-18	0	1	1	0	I	0	58	Female
NV-19	0	1	1	0	0	0	54	Female
NV-20	0	1	0	1	I	I	54	Male
NV-21	0	0	0	0	0	0	69	Female
NV-22	0	0	0	0	0	0	71	Female
NV-23	0	0	0	0	0	0	44	Female
NV-24	0	0	0	1	I	I	49	Female
NV-25	0	0	0	0	I	0	56	Male
NV-26	0	0	0	0	I	I	58	Male
NV-27	0	1	1	0	I	0	48	Female
NV-28	0	1	1	0	I	0	51	Female
NV-29	0	1	1	1	0	0	70	Male
NV-30	0	1	1	0	0	0	65	Male

The table specifies the most relevant clinical characteristics from the endoscopy and pathology reports for each patient, including gastric cancer status, Rapid Urease Test (RUT) result, *H. pylori* and intestinal metaplasia in the pathology report, and OLGA and OLGIM status. Information regarding age and sex is also indicated. 0 and 1 indicate negative and positive, respectively, for gastric cancer, RUT, *H. pylori* and intestinal metaplasia in the pathology report.

**Figure 5:**
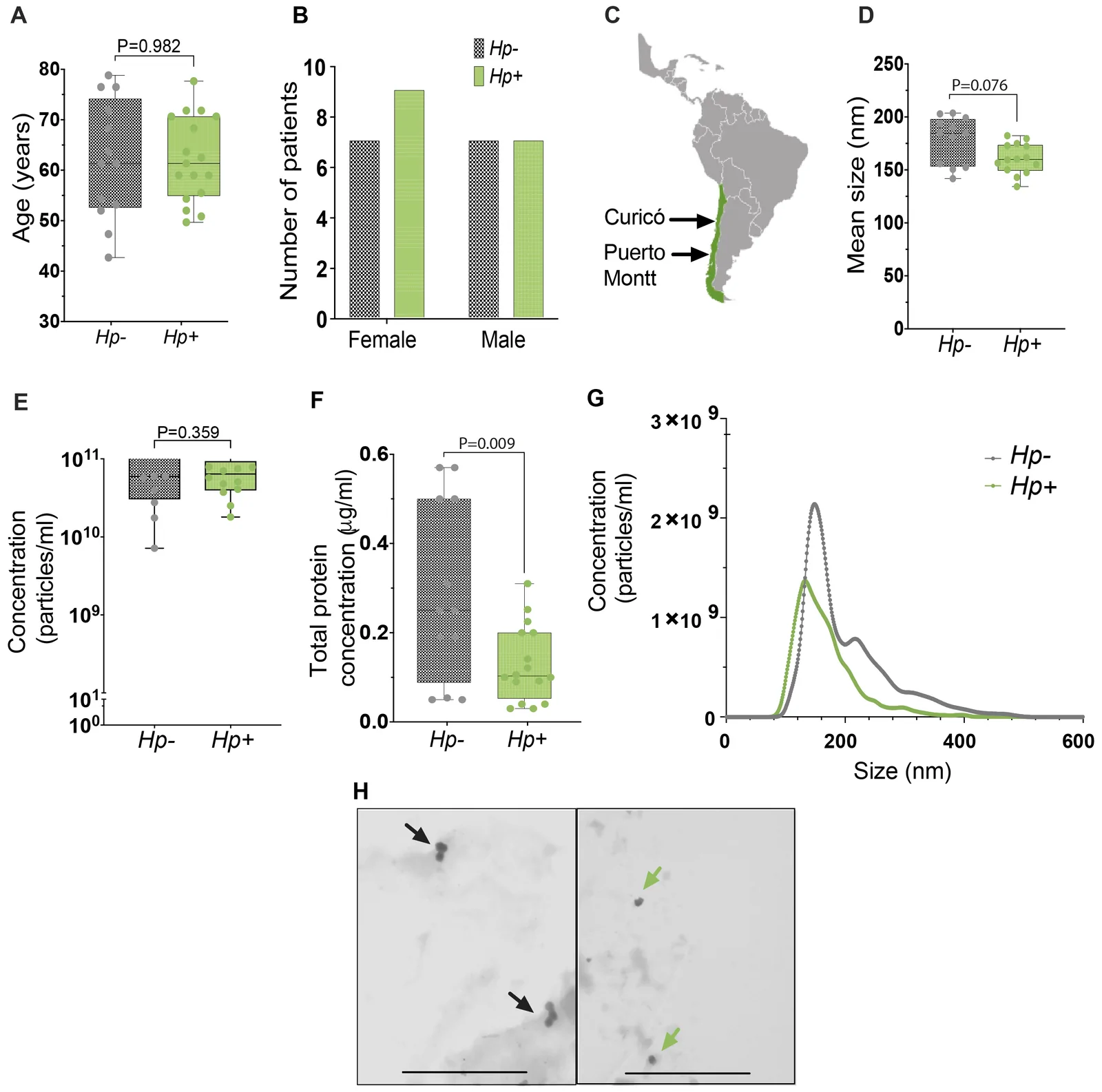
Demographic characteristics and characterization of EVs purified from patient-derived serum. **(A)** mean age in the *Hp-* and *Hp+* groups. **(B)** number of *Hp-* and *Hp+* female and male patients, **(C)** geographical location of the patients included in the study. EVs were isolated from the serum of thirty patients and then characterized by NTA, total protein concentration quantification, and scanning electron microscopy (SEM), obtaining the **(D)** mean size, **(E)** vesicle concentration, **(F)** total protein concentration, **(G)** the NTA profile (vesicle concentration vs. size), and **(H)** SEM to corroborate the shape and size of the EVs from the *Hp-* (left panel) and *Hp+* (right panel) groups. Arrows in **(H)** indicate the EVs. Magnification bar = 2 µm. Statistical analyses were performed using t-test. Exact *p* values are reported in the figure.

### 3.4 Characterization of serum-derived EVs in *H. pylori* infected and non-infected patients

To isolate and characterize the serum-derived EVs from patients, we employed our optimized protocol for the purification of EVs summarized in **Figure 4A** (see Methods section for more details). EVs were purified from thirty patients, stratified into fourteen *H. pylori-*negative (*Hp*-) and sixteen *H. pylori-*positive (*Hp+*) groups. NTA characterization of the EVs revealed that neither their average size (**Figure 5D**) nor the particle concentration (**Figure 5E**) was different between the two groups. The quantification of total protein concentration revealed that EVs from *Hp+* patients exhibited a lower total protein concentration than those from *Hp-* patients (**Figure 5F**). Representative NTA profile also indicated a consistent size distribution of EVs between groups (particle concentration vs. size), with a peak around 100–150 nm range, typical of EVs (**Figure 5G**). Finally, the EVs were analyzed using scanning electron microscopy (SEM), validating their characteristic vesicular rounded shape and size (**Figure 5H**). The average size of EVs as measured by SEM was 150 ± 7 nm for *Hp*- and 129 ± 9 nm for *Hp*+ samples (**Figure 5H**), indicating a trend toward slightly smaller vesicles in infected individuals. The reduced size observed in SEM relative to NTA data is likely attributable to the dehydration of vesicles generated by the sample fixation protocol used for such analysis, as previously reported (Théry et al., 2018).

### 3.5 Nanogram immunodetection of *H. pylori* Urease in EVs from human serum

Urease is one of the most abundant peptides in purified Hp-EVs (Palacios et al., 2023), and our findings indicate that, among several virulence factors tested, UreB is the one detected at the lowest amount of Hp-EVs, becoming visible by IB at ~50 ng (**Figure 1**). Therefore, we utilized the detection of Urease, employing a specific antibody to detect UreB from *H. pylori* in the EVs isolated from serum, as an indicator of their presence in human serum and evidence of *H. pylori* infection. The results show that *H. pylori* UreB was detected in 62.5% of the patients who were considered true positive for *H. pylori* (**Figures 6A, B**). The EV markers CD63 and CD81 were detected in the serum-derived EVs from all patients (*Hp*+ and *Hp*-), confirming the reliability of the purification procedure and the functionality of the detection method (**Figure 6A**). Noteworthy, the detection of EVs positive for UreB by IB provides the first evidence for circulating Hp-EVs in human blood.

**Figure 6:**
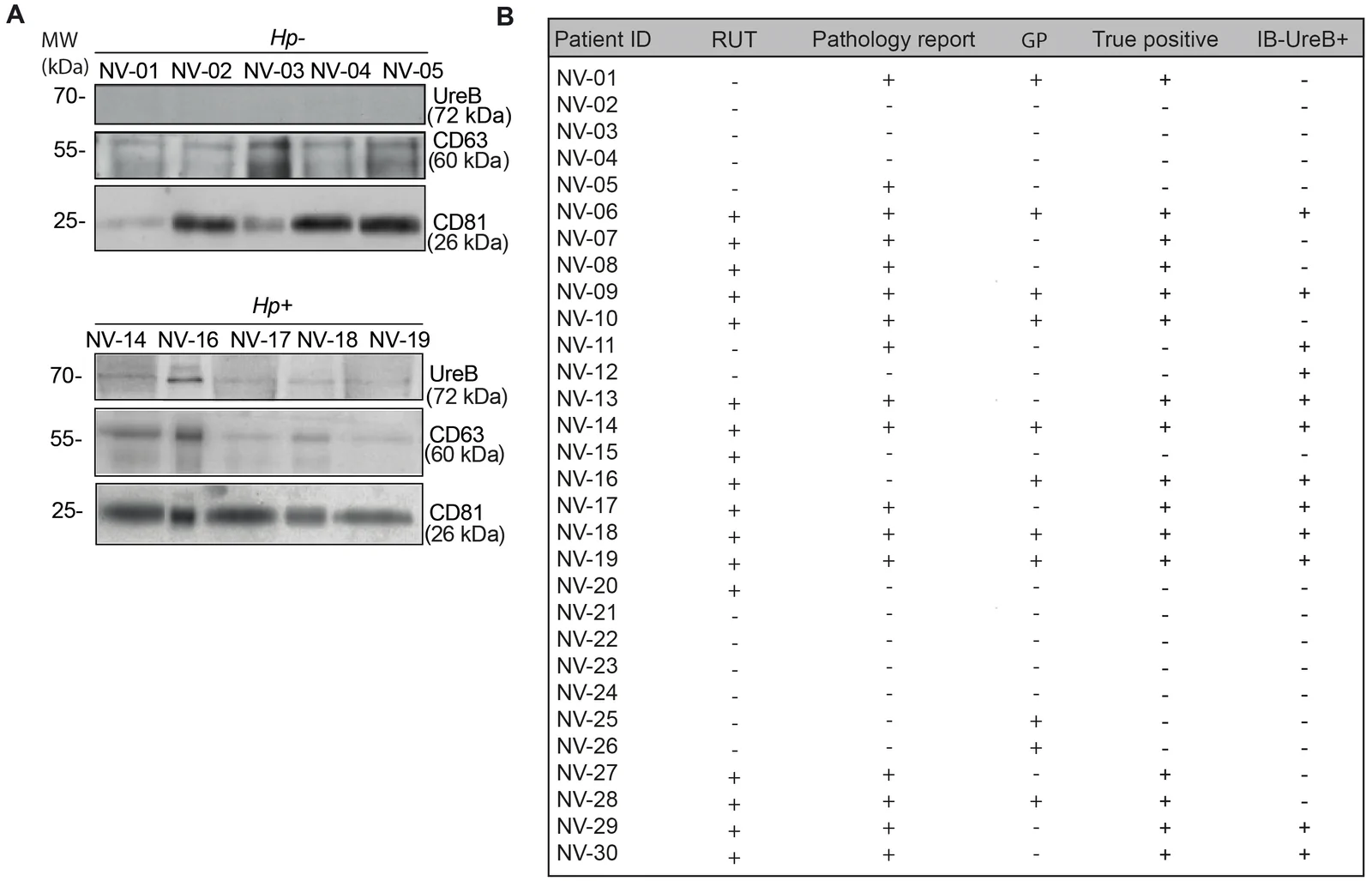
Urease immunodetection in the EVs from the serum of patients infected with *H. pylori*. **(A)** A representative image of the immunoblots obtained for the EVs purified from the serum of the thirty patients to detect the presence of *H. pylori* Urease and the CD63 and CD81 EV markers using specific antibodies*. Each line represents a different patient, whose code is indicated in the image, Molecular weight (MW) values in kDa are indicated to the left. In **(B)**, we show a table that compares the results obtained from the detection of *H. pylori* Urease by immunoblot (IB-UreB+) and those reported by RUT and the histopathology report of the gastric biopsy. In addition to the result of GP, used as a third reference test, to resolve discordant cases, resulting in the true positive cases. * Uncropped IBs are in Supplementary file.

### 3.6 GastroPanel® (GP)

To further characterize the serum of patients included in this study, a series of commercially available gastric health tests based on four biomarkers called *GastroPanel*® (GP) were performed, which measures the Pepsinogen I/II ratio, Gastrin17, and anti-*H. pylori* antibodies (anti-Hp antibodies). The results of the GP tests are shown in **Table 2**. The Pepsinogen I/II ratio serves as an effective predictor of gastric lesions, with levels equal to or below 3.0 (≤ 3.0) indicating a positive correlation with the presence of such lesions. On the other hand, the secretion level of gastric acid strongly correlates with the serum concentration of the gut-derived peptide hormone Gastrin17 (Wang et al., 2021). *H. pylori* infection can modulate acid secretion, either increasing or decreasing it, depending on several factors (Schubert, 2017); therefore, abnormal Gastrin17 levels, outside its normal range of 1.8–7 may suggest an infection with the bacterium. Finally, the result of the detection of anti-*H. pylori* antibody is considered positive when the level is above 25, consequently, according to this test, only eleven patients had circulating antibodies for *H. pylori*.

**Table 2:** *GastroPanel^®^* (GP) performed on the sera of the thirty patients included in the study.

ID	PGI/PGII	Gastrin17	*Anti-Hp antibodies*
NV-01	4.69	> 34.7*	54.25*
NV-02	1.33*	> 34.7*	< 10.6
NV-03	6	1.94	< 10.6
NV-04	3.85	5.53	19.1
NV-05	8.6	9.37*	14.36
NV-06	4.74	5.93	85.27*
NV-07	5.09	8.48*	< 10.6
NV-08	4.6	3	< 10.6
NV-09	2.0*	32.99*	> 242*
NV-10	4.84	8.35*	> 242*
NV-11	0.66*	> 34.7*	< 10.6
NV-12	8.96	20.34*	< 10.6
NV-13	7.77	4.95	< 10.6
NV-14	5.06	1.88	51.13*
NV-15	6.04	4.58	< 10.6
NV-16	9.4	< 1.0*	55.44*
NV-17	5.8	17.3*	< 10.6
NV-18	8.72	1.75*	75.47*
NV-19	7.81	1.68*	38.83*
NV-20	14.58	27.07*	< 10.6
NV-21	5.08	2.22	< 10.6
NV-22	10.76	4.21	< 10.6
NV-23	8.79	4.91	10.97
NV-24	10.38	7.61	< 10.6
NV-25	9.52	7.92	67.11*
NV-26	9.54	1.19*	36.78*
NV-27	4.93	4.24	< 10.6
NV-28	3.41	9.19	> 242*
NV-29	7.05	1.61*	< 10.6
NV-30	6.28	< 1.0*	< 10.6

The table shows the results of complementary commercially available clinical tests related to gastric health, which were performed on patient serum. The GP detects PepsinogenI/PepsinogenII (PGI/PGII), Gastrin17, and anti-Hp antibodies. The asterisk indicates an abnormal value.

### 3.7 Diagnosis of *H. pylori* infection based on immunodetection of *H. pylori* Urease-positive vesicles

Since the diagnosis of *H. pylori* infection has been primarily based on invasive procedures, which implies discomfort and financial burden for patients, we assessed whether the detection of *H. pylori* UreB in serum-derived EVs by IB (IB-UreB+) could serve as an efficient, minimally invasive, with limited discomfort or risk, and cost-effective alternative for a primary diagnosis of ongoing infections. Then, using univariate and multivariate logistic regression, we assessed the ability of the IB-UreB^+^ assay to accurately identify these confirmed cases. The predictive power of the IB-UreB+ test was contrasted in terms of sensitivity, specificity, positive and negative predictive power, and accuracy against the GP (which is also a minimally invasive method) and each one of their biomarkers separately. For the comparison with GP, we converted the clinical reports generated by qualified healthcare professionals into a binary classification system, where positive results were coded as 1 and negative results as 0. When comparing against individual biomarkers, we similarly employed a binary classification approach based on manufacturer-established reference values, designating normal values as 0 and abnormal values as 1. Ultimately, we evaluated whether incorporating the IB-UreB+ assay as a supplementary serum diagnostic biomarker could enhance the precision of the GP test, when integrated into a multivariate predictive model. In **Table 3**, we show that, among predictors, the IB-UreB+ assay emerged as the strongest predictor with a significant odds ratio (OR = 10.00, IC95% = 1.899, 80.35, P=0.0125*), demonstrating a robust association with the outcome variable. Additionally, anti-Hp antibodies, GP, Pepsinogen I/II ratio and Gastrin17 reported odds ratios slightly above 1. This association did not reach statistical significance, likely due to limitations imposed by the small sample size (n = 30). Consistent with these findings, receiver operating characteristic (ROC) curves derived from univariate logistic regression analyses (**Figures 7A–D**) revealed that IB-UreB+ data generated the highest area under the ROC curve (AUC = 0.74 ± 0.09) outperforming GP (AUC = 0.64 ± 0.10, **Figure 7A**) and representing the only individual marker with a statistically significant improvement over the intercept-only model. In contrast, the Pepsinogen I/II ratio (AUC = 0.53 ± 0.10, **Figure 7B**), anti-Hp antibodies (AUC = 0.64 ± 0.10, **Figure 7C**) and Gastrin17 (AUC = 0.56 ± 0.10, **Figure 7D**) did not improve discrimination relative to GP. When tested against the intercept-only model, only IB-UreB+ assay showed statistically significant improvement in diagnostic performance (P= 0.024). The combined model of GP and IB-UreB+ assays (multivariate model, **Figure 7E**) achieved a superior AUC (0.80 ± 0.09) than IB-UreB+ (P= 0.006). Notably, the addition of IB-UreB+ increased the predictive power of GP, acting as a convenient complementary biomarker. Along this line, diagnostic performance evaluation revealed that the combined GP + IB-UreB+ model achieved superior performance on sensitivity (81.25%, **Figure 7F**), specificity (71.43%, **Figure 7G**), positive predictive power (PPV, 76.47%, **Figure 7H**), negative predictive power (NPV, 76.92%, **Figure 7I**), and accuracy (76.67%; **Figure 7J**). The IB-UreB+ test alone demonstrated the second-best performance, exhibiting moderate sensitivity (62.50%, **Figure 7F**) and NPV (66.67%, **Figure 7I**), but high specificity (85.71%, **Figure 7G**) and PPV (83.33%, **Figure 7H**), yielding an overall accuracy of 73.33% (**Figure 7J**). GP and anti-Hp antibodies showed equivalent performance, ranking third with moderate specificity (78.57%, **Figure 7G**) and PPV (72.73%, **Figure 7H**) but suboptimal sensitivity (50.00%, **Figure 7F**), NPV (57.89%, **Figure 7I**), and accuracy (63.33%, **Figure 7J**). Gastrin17 and Pepsinogen I/II ratio exhibited the poorest diagnostic performance, with sensitivities of 68.75% and 12.50% (**Figure 7F**), respectively. Notably, the Pepsinogen I/II ratio’s apparent high specificity (92.86%, **Figure 7G**) likely represents a statistical artifact due to limited sensitivity coupled with small sample size, resulting in poor overall diagnostic utility (accuracy 50.00%, **Figure 7J**). Model selection analysis using the corrected Akaike Information Criterion (AICc, **Figure 7K**) revealed a clear performance hierarchy among the evaluated predictors. The IB-UreB+ assay emerged as the superior model (ΔAICc = -5.43), followed by the combined GP + IB-UreB+ approach (ΔAICc = -4.39). Both models exhibited substantial negative ΔAICc values, indicating strong statistical support and a superior fit compared to the intercept-only baseline model. In contrast, GP and Anti-Hp antibodies demonstrated equivalent but marginal performance (both ΔAICc = -0.40), suggesting minimal improvement over the null model. On the other hand, Gastrin17 and Pepsinogen I/II ratio showed the poorest performance with positive ΔAICc values (1.87 and 2.06, respectively), indicating that these models provide insufficient explanatory power. Thus, the information-theoretic framework establishes IB-UreB+ assay and the integrated GP + IB-UreB+ model as the only statistically justified approaches for *H. pylori* detection, with all other predictors failing to demonstrate adequate model support for clinical diagnostic applications.

**Table 3:** Logistic regression models evaluating the diagnostic performance of different serum biomarkers for *H. pylori* infection across several univariate and multivariate models.

Model	Variable	β coefficient	95% CI	OR	95% CI	*P* value	AICc	Log-likelihood ratio (G squared)	*P* value
*IB-UreB+*	Intercept (β0)	-0.6931	-1.748, 0.2527	0.5000	0.1741, 1.287	0.1657	43.60	7.727	0.0054**
	IB UreB+ (β1)	2.303	0.6413, 4.386	10.00	1.899, 80.35	0.0125*	38.17		
*GP*	Intercept (β0)	-0.3185	-1.267, 0.5859	0.7273	0.2816, 1.797	0.4931	43.60	2.701	0.1003
	GP (β1)	1.299	-0.2433, 3.049	3.667	0.7840, 21.09	0.1136	43.20		
*PGI/PGII*	Intercept (β0)	0.07411	-0.6862, 0.8416	1.077	0.5035, 2.320	0.8474	43.60	0.2434	0.6218
	PGI/PGII (β1)	0.6190	-1.838, 3.756	1.857	0.1592, 42.78	0.6297	45.66		
*Anti-Hp antibodies*	Intercept (β0)	-0.3185	-1.267, 0.5859	0.7273	0.2816, 1.797	0.4931	43.60	2.701	0.1003
	Anti-Hp antibodies (β1)	1.299	-0.2433, 3.049	3.667	0.7840, 21.09	0.1136	43.20		
*Gastrin17*	Intercept (β0)	-0.1823	-1.426, 1.018	0.8333	0.2402, 2.767	0.7633	43.60	0.4333	0.5104
	Gastrin17 (β1)	0.5008	-0.9947, 2.040	1.650	0.3698, 7.691	0.5118	45.47		
*GP +* *IB-UreB+*	Intercept (β0)	-1.027	-2.337, 0.07320	0.3581	0.09665, 1.076	0.0858	43.60	9.167	0.0102*
	GP (β1)	1.085	-0.6898, 3.011	2.960	0.5017, 20.31	0.2373	39.21		
	IB UreB+ (β2)	2.191	0.4793, 4.308	8.946	1.615, 74.26	0.0200			

The models include IB-UreB+, GP, PGI/PGII ratio, anti-Hp antibodies, Gastrin17, and a combined model of GP with IB-UreB+. For each model, the table reports regression coefficients (B) with 95% confidence intervals, odds ratios (OR) with corresponding 95% confidence intervals, *P* values, corrected Akaike information criterion (AICc), log-likelihood ratio, and Hosmer-Lemeshow goodness-of-fit test (G-squared) with *P* values. For each model, the comparison performed by Akaike information criterion (AICc) and log-likelihood ratio were contrasting intercept-only-model versus selected model (all variables including the intercept). * *P* ≤ 0,05; ** *P* ≤ 0,01.

**Figure 7:**
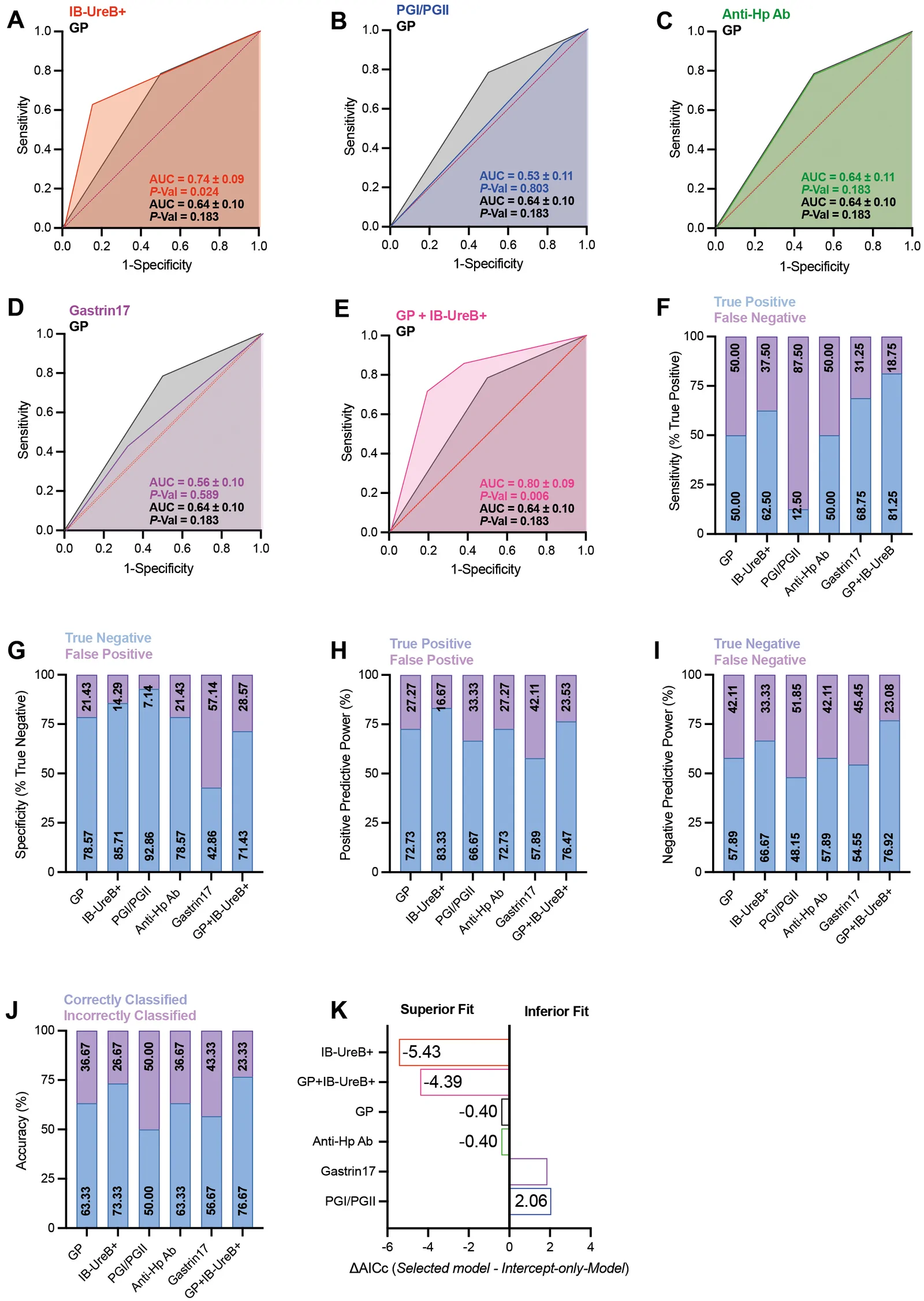
Diagnostic performance of IB-UreB+ in Extracellular Vesicles versus conventional *H. pylori* detection methods. **(A–E)** ROC curves from univariate and multivariate logistic regression showing discriminatory performance of GP vs **(A)** IB-UreB+, **(B)** PGI/PGII ratio, **(C)** Anti-Hp antibodies (Anti-Hp Ab), **(D)** Gastrin17, and **(E)** GP+IB-UreB+ combination, with respective AUC values, standard deviation, and P-Value contrasting against the intercept-only model (random classification of cases). F-J, Quantitative assessment of diagnostic parameters: **(F)** sensitivity, **(G)** specificity, **(H)** positive predictive power, **(I)** negative predictive power, and **(J)** overall accuracy. **(K)**, Model quality evaluation using ΔAICc values relative to the intercept-only model, with negative values indicating superior predictive performance. IB-UreB+ alone and in combination with GP demonstrated the strongest statistical support.

These findings suggest that IB-UreB+ consistently retains its diagnostic value as assessed by different parameters and that performance could be enhanced when integrated with GP results. Therefore, this combined strategy appears as a potential minimally invasive approach to detect *H. pylori* infection using patient serum samples.

## 4 Discussion

Here, we optimized a size exclusion chromatography-based protocol for the purification and detection of Hp-EVs circulating in the blood of patients infected with *H. pylori*, thereby: 1) providing for the first time direct evidence of the presence of Hp-EVs in human circulation and, 2) proposing their detection in blood as a potential minimally invasive approach to identify patients with current *H. pylori*-positive status.

Hp-EVs isolated from *H. pylori* liquid cultures allowed us to assess the most abundant virulence factors present in these bacterial vesicles by IB. Urease was the best antigen to detect the presence of these EVs, being quantifiable at the nanogram scale in terms of the total protein content (**Figure 1**). These results are consistent with other *in vitro* studies reporting that Urease is one of the most abundant virulence factors in Hp-EVs (Zavan et al., 2019; Palacios et al., 2023). The presence of Urease in Hp-EVs has gained considerable attention in recent years as this enzyme has been implicated as a key factor in increasing endothelial permeability through mechanisms that remain poorly understood but appeared to be independent of its enzymatic activity (de Jesus Souza et al., 2019). Likewise, it has been suggested that the Urease could act as a ligand for the toll-like receptor 2 (TLR2) present in the membrane surface of several cell types (Koch and Müller, 2015; Valenzuela-Valderrama et al., 2019). Such Urease-TLR2 interaction would trigger a proinflammatory response via NF-κB that can contribute to the development or worsening the extra-gastric pathologies associated with *H. pylori* infection (Park and Tsunoda, 2022; Uberti et al., 2022; Palacios et al., 2023). Noteworthy, the abundance of virulence factors of the Hp-EVs described here differs significantly from other studies, where CagA has been reported as the only biomarker in human serum-derived EVs detected from patients with *H. pylori* infection (Shimoda et al., 2016). CagA is a virulence factor known to be “injected” into the host gastric epithelial cells via the bacterial type IV secretion system (T4SS). Therefore, the CagA detected in EVs purified from patient serum by Shimoda and coworkers (Shimoda et al., 2016) is most likely present in h-EVs rather than Hp-EVs. Thus, we report here for the first time, evidence of the presence of Hp-EVs in the human bloodstream, providing direct evidence for their systemic dissemination during infection. In contrast to our findings, Shimoda et al. did not report Urease in the serum-derived EVs from patients infected with *H. pylori*. This discrepancy may be attributable to methodological differences including different mass spectrometry analyses, different EV purification methods, or differences in the clinical o physiological conditions of the patient cohorts. In support of this view, our results align with previous reports (Olofsson et al., 2010; Zavan et al., 2019; Palacios et al., 2023), which consistently identify Urease as one of the most abundant virulence factors in Hp-EVs. Since bacterial EVs account only for a small fraction of the total EVs isolated from serum, it is also likely that a more sensitive method is required to detect bacterial EVs, i.e., IB.

A limitation of the present study is that the bacterial origin of the detected UreB-positive vesicles was inferred from immunodetection rather than confirmed using independent analytical approaches. Additional characterization of the vesicular fraction by proteomic profiling, immunogold electron microscopy for UreB, or detection of *H. pylori*-specific membrane markers would further strengthen this assignment. Nevertheless, several aspects of our experimental workflow support the interpretation that the detected UreB signal is associated with *H. pylori*-derived vesicles. EVs were isolated by size-exclusion chromatography, a method that efficiently separates vesicles from most soluble proteins based on size, with vesicle-enriched fractions eluting separately from free proteins (Benedikter, et al. 2017; Böing, et al. 2014). In addition, serum samples were centrifuged and filtered through 0.22 μm membranes before isolation, substantially reducing the presence of bacterial debris and other large particulate contaminants. Furthermore, although we cannot completely exclude the possibility that some UreB may be associated with h-EVs following uptake of bacterial material, Urease has not been reported as a major cargo of h-EVs during *H. pylori* infection. Taken together, these considerations support the interpretation that the detected UreB signal originates predominantly from bacterial vesicles.

Another limitation of this study is that all serum samples had been stored at –80 °C for approximately five years prior to vesicle isolation and UreB immunodetection. Long-term storage may affect vesicle integrity, protein stability, and consequently the sensitivity of biomarker detection. Nevertheless, EVs were reproducibly recovered from all samples and exhibited the expected size distribution by NTA, characteristic vesicular morphology by SEM, and consistent expression of the EV markers CD63 and CD81. These results suggest that vesicle integrity was largely preserved under the storage conditions employed and indicate that archived bio-banked samples can serve as valuable resource for exploratory EV-based studies. However, because matched fresh samples were not available, the effect of long-term storage on UreB detectability could not be directly evaluated. Storage-related degradation may have reduced signal intensity and led to an underestimation of UreB positivity. Therefore, future prospective studies comparing fresh and long-term stored samples are needed to assess the impact of storage on assay performance and validate its clinical applicability.

Using our optimized protocol (**Figure 4A**), we purified the serum-derived EVs from thirty patients, fourteen negative and sixteen positives for *H. pylori* infection (**Table 1**). Notably, NTA and SEM analyses showed that *Hp+* patients exhibited a tendency to smaller vesicles than *Hp-* individuals (**Figures 5D, H**). These results are similar to others obtained in an *in vitro* model, where gastric cells infected with *H. pylori* release EVs with a mean size significantly smaller than gastric cells without *H. pylori* infection (González et al., 2022). The *Hp-* and *Hp+* patient groups did not differ in age or sex distribution (**Figure 5A, B**), indicating that these demographic variables are unlikely to confound the observed differences in circulating EVs. Such findings support the idea that *H. pylori* infection shifts the EV population in circulation toward vesicles influenced by bacterial components or infection-induced host responses. In contrast, the total protein content of EV preparations, often used as an indicator of EV abundance, was significantly lower in *Hp+* relative to *Hp-* samples (**Figure 5F**). These results appear to contradict reports that EVs are more abundant in circulation, in certain pathological contexts, including pathogenic infections, inflammation, and cancer (Sellam et al., 2009; Baran et al., 2010; Taxiarchis et al., 2024). However, several factors could reconcile this discrepancy. First, the average age of the patient cohort was 57 years, an age at which systemic EV homeostasis is known to change. Aging is associated with altered EV biogenesis, increased phagocytic clearance by immune cells, and shifts in EV subtype composition. These age-related processes can result in overall lower EV concentrations in circulation, even in the presence of pathological stimuli (Liu et al., 2020). Second, smaller vesicles often carry less protein per particle, despite unaltered or even increased particle numbers (Zendrini et al., 2022). Third, the inflammatory profile associated with chronic *H. pylori* infection can modulate EV turnover rates *in vivo*, including enhanced uptake and degradation by macrophages and endothelial cells (Turner et al., 2018; Jin et al., 2022). Together, these factors reflect a complex interplay among infection status, aging-associated changes in EV clearance, and shifts in vesicle subtype production. These findings underscore the importance of analyzing EV size, concentration, and composition, rather than relying solely on total protein measures, to accurately interpret EV dynamics in infected individuals. Given the observed differences in the size and protein content of EVs in *Hp*+ individuals, the potential of these vesicles as biomarkers warranted further consideration. To contextualize their diagnostic value, it is important to compare current *H. pylori* detection strategies—particularly those considered non-invasive—in terms of sensitivity, specificity, and clinical applicability as minimally invasive tools.

Building on our findings, the combination of IB-UreB+ and the GP—both performed from a minimally invasive blood sample—demonstrated high sensitivity and specificity for detecting *H. pylori* infection. Although serum IB-UreB+ test demonstrated moderate sensitivity (ROC, 62.5%) in detecting patients with confirmed *H. pylori* infection by dual standard reference (RUT and Pathologist report, both from gastric biopsies), their diagnostic potential surpassed that of the clinically established GP. The IB-UreB+ assay exhibited significantly higher predictive power for *H. pylori* infection diagnosis with superior sensitivity, specificity, positive predictive power, negative predictive power, and accuracy, compared to GP. The GP was the first minimally invasive diagnostic tool to detect three physiological stomach-specific biomarkers (Gastrin17, Pepsinogen I and II) and anti-Hp antibodies by ELISA (Syrjänen et al., 2019). This tool provides information about stomach health by testing some of the increased risk factors for stomach and esophageal cancer, as well as for disorders of malabsorption due to atrophic gastritis. Hence, it is not surprising that several studies have supported their use for the diagnosis of *H. pylori* infection as well as other stomach pathologies (Syrjänen, 2016; Syrjänen et al., 2019). However, the use of these biomarkers for diagnosis of *H. pylori* infection has shown controverted results because Gastrin17 and Pepsinogens are biomarkers for tissue damage (He et al., 2011; Kikuchi et al., 2011; Shen et al., 2021), and the only biomarker directly related to *H. pylori* infection is the *H. pylori* antibody detection. However, this test can be affected by a variety of factors, including the *H. pylori* load, the natural loss of infection due to advanced atrophy, or a false-positive serology due to previous infection (Syrjänen, 2016; Kayali et al., 2018). In fact, when the efficiency of GP was evaluated in dyspeptic patients, the test showed a high sensitivity for atrophic gastritis (82.9%) but a low sensitivity for the diagnosis of *H. pylori* infection (53.8%) (Masci et al., 2014), which is in line with the limitations of using anti-Hp antibody detection as a biomarker. Similar results were reported in another study on children with *H. pylori* infection, where they found that none of the biomarkers included in GP are sensitive enough for *H. pylori* screening and do not replace endoscopy, at least for diagnosis in children (Koivusalo et al., 2007). Several studies have reported that the next generation of GP demonstrates enhanced sensitivity compared to previous versions, with *H. pylori* antibody detection reaching sensitivity values above 90% (Mäki et al., 2020; Koivurova et al., 2021; Paloheimo et al., 2021). Nevertheless, in our study, the IB-UreB+ test showed a higher sensitivity than that of anti-Hp antibodies, which was 50%, consistent with previously reported findings in pediatric and adult dyspeptic patients (Koivusalo et al., 2007; Masci et al., 2014).

On the other hand, the moderate sensitivity observed for the IB-UreB+ test is likely due to the detection method used—Immunoblot—which is inherently less sensitivity than the ELISA format employed in the GP assay. Therefore, developing an ELISA kit specifically designed to detect UreB+ EVs would likely improve sensitivity and strengthen its value as a serum biomarker for *H. pylori* infection.

Accordingly, discrepancies between RUT and IB-UreB results are not unexpected because the two assays measure different properties of Urease in distinct biological compartments. RUT measures Urease enzymatic activity in gastric biopsy tissue, whereas IB-UreB detects the presence of UreB protein in vesicles isolated from blood, regardless of its enzymatic activity. As a result, differences between the two methods may arise from biological differences between the stomach and bloodstream, the lower sensitivity of immunoblotting compared to enzymatic detection, or variability among patients in the release of UreB-containing vesicles into systemic circulation. Therefore, IB-UreB should be viewed as a minimally invasive blood-based biomarker that complements, rather than replaces, gastric diagnostic tests such as RUT.

To explore potential factors contributing to false-negative IB-UreB results, we analyzed the available clinicopathological data to determine whether UreB detectability was associated with the severity of gastric lesions. No significant relationship was observed between UreB positivity and disease severity as assessed by either OLGA (P= 0.266) or OLGIM (p > 0.999) staging systems. However, these findings should be interpreted with caution, as the limited sample size and the asymmetric distribution of patients across disease stages likely limit the statistical power to detect meaningful associations. Consequently, we cannot exclude the possibility that disease severity influences the release of UreB+ vesicles into the bloodstream or their detectability in serum.

A positive aspect for the intended use of UreB+ EVs as a diagnostic method is that EVs have a much shorter half-life in the bloodstream compared to antibodies (Matsumoto et al., 2021; Figueroa Villalba et al., 2024; Heidinger et al., 2024). As a result, they disappear from circulation soon after infection is cleared. In contrast, anti-Hp antibodies can remain detectable for up to six months after successful eradication. This difference suggests that the detection of UreB+ EVs may be more closely associated with the presence of an ongoing *H. pylori* infection, whereas antibody-based tests can remain positive long after bacterial eradication and may therefore reflect past infection rather than current bacterial presence. However, because we did not evaluate post-eradication samples or assess potential cross-reactivity with other bacterial infections, the ability of UreB+ EVs to specifically identify ongoing *H. pylori* infection remains to be established. Therefore, UreB+ EVs should currently be regarded as a promising candidate biomarker associated with the presence of *H. pylori*-derived products in serum, rather than as a validated marker of active infection. Longitudinal studies including pre- and post-eradication samples will be required to determine whether this biomarker can reliably distinguish ongoing infection from past exposure.

One of the main limitations of this exploratory study is the small sample size (n= 30). This may explain the discrepancies we observed in GP performance compared with recently published studies (Mäki et al., 2020; Paloheimo et al., 2021). However, we must emphasize that our intention is not to discourage the use of GP or to downplay its value in diagnosing *H. pylori* infection. Instead, our primary objective was to demonstrate that Hp-EVs are present in the bloodstream of infected patients. Their detection supports the hypothesis that these Hp-EVs can cross biological barriers, such as epithelial and endothelial layers, which may help explain how this bacterium contributes to extra-gastric effects. Furthermore, detecting bacterial EVs in serum highlights their potential as minimally invasive biomarkers of *H. pylori* infection. Although this approach may reduce the need for invasive and expensive diagnostic procedures, such as gastric biopsy or endoscopy, larger prospective studies will be needed to confirm its diagnostic performance and clinical utility.

In conclusion, the detection of Hp-EVs circulating in the blood of infected patients stands as a pivotal finding. It additionally reveals two other critical insights: i) Hp-EVs can cross epithelial barriers, offering a plausible mechanism for the bacterium’s extra-gastric effects, and ii) these systemic UreB+ EVs therefore represent a promising candidate biomarker associated with current *H. pylori* positive status.

## 5 Conclusions

An optimized strategy for the isolation and characterization of EVs from human serum enabled us to detect *H. pylori*-derived (UreB+) EVs in circulation. Such identification of UreB+ EVs by IB provides, to our knowledge, the first evidence that Hp-EVs circulate in the blood of infected individuals, supporting a mechanism by which bacterial virulence factors may disseminate systemically beyond the gastric niche. Furthermore, combining UreB detection with the GP test improved diagnostic performance, highlighting the potential of this integrated approach as a minimally invasive strategy for detecting *H. pylori* infection in patient blood samples.

## Data Availability

The original contributions presented in the study are included in the article/**Supplementary Material**. Further inquiries can be directed to the corresponding authors.
