# Supplementary material for: Circulating Helicobacter pylori extracellular vesicles in serum as biomarkers of infection
Source: Front Cell Infect Microbiol. 2026 Sep 16;16:1893888. doi: 10.3389/fcimb.2026.1893888 (PMC13623701; doi:10.3389/fcimb.2026.1893888)
Supplement: Supplementary file 1 [file DataSheet1.pdf]

Figure 1 (Original immunoblot). The section framed in the red box was the one used for Figure 1. It was done this way so that the lane of the *Helicobacter pylori* bacterial lysate, which was used as a positive control, would not be saturated.

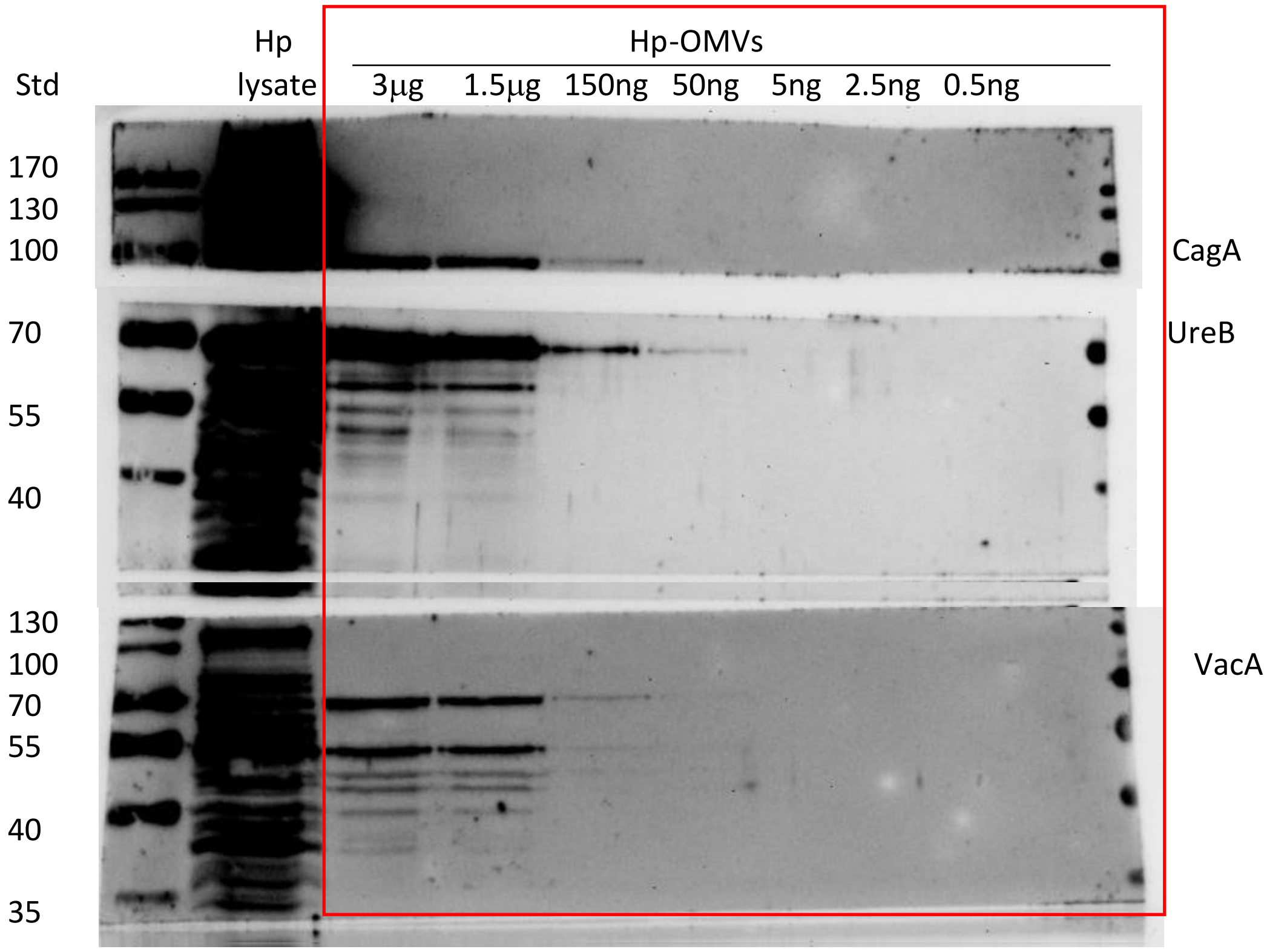

Photograph of the membrane, in which the molecular weight standard can be observed

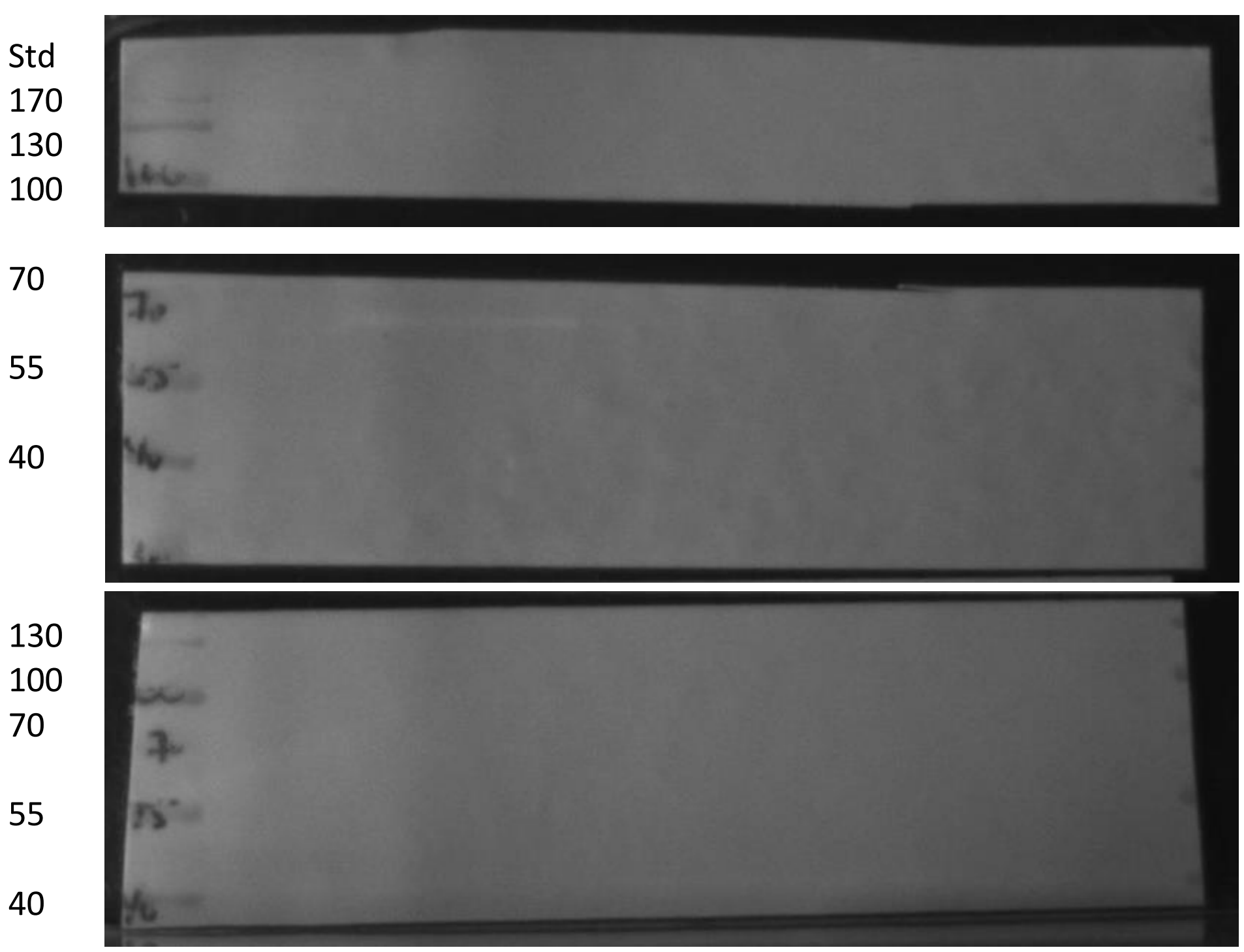

Figure 3D (Original immunoblot). The final photo was cropped and rearranged so that the order of the wells matched the rest of the graphs in Figure 3.

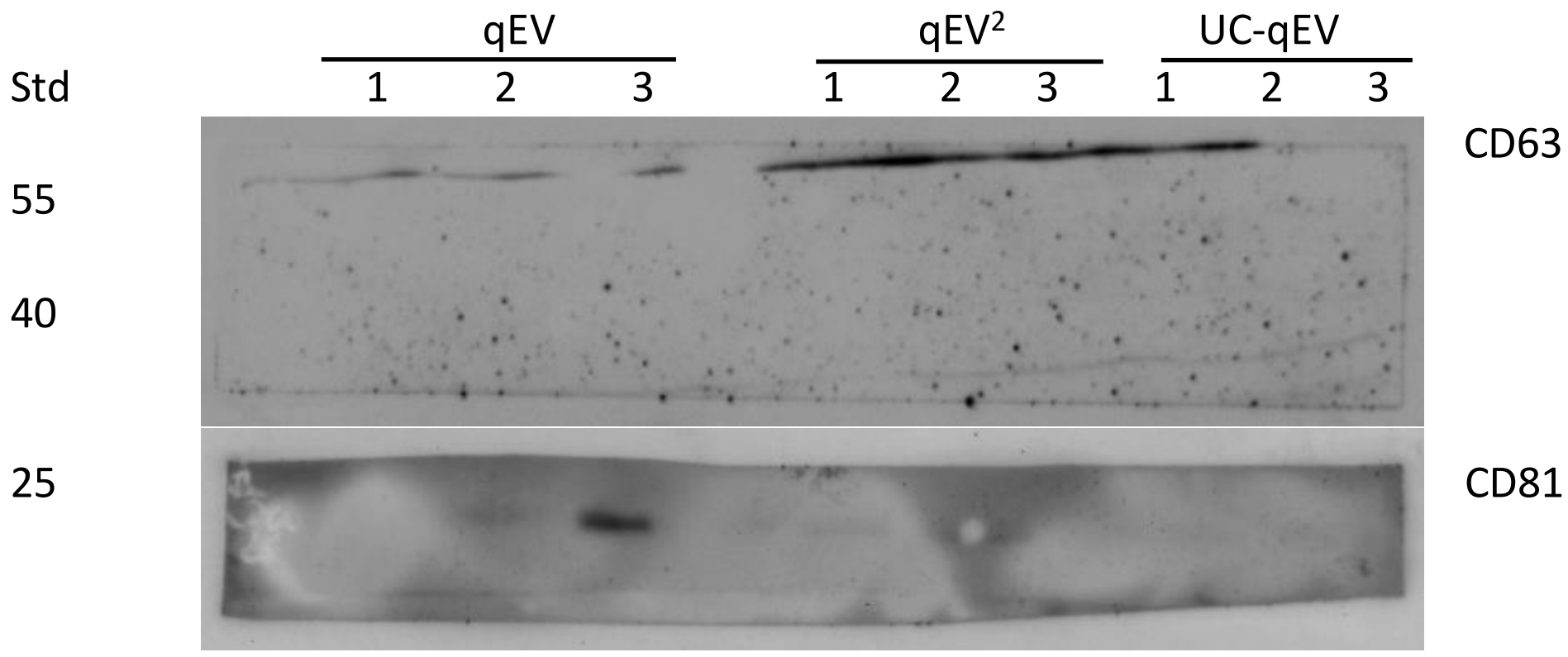

Photograph of the membrane, in which the molecular weight standard can be observed

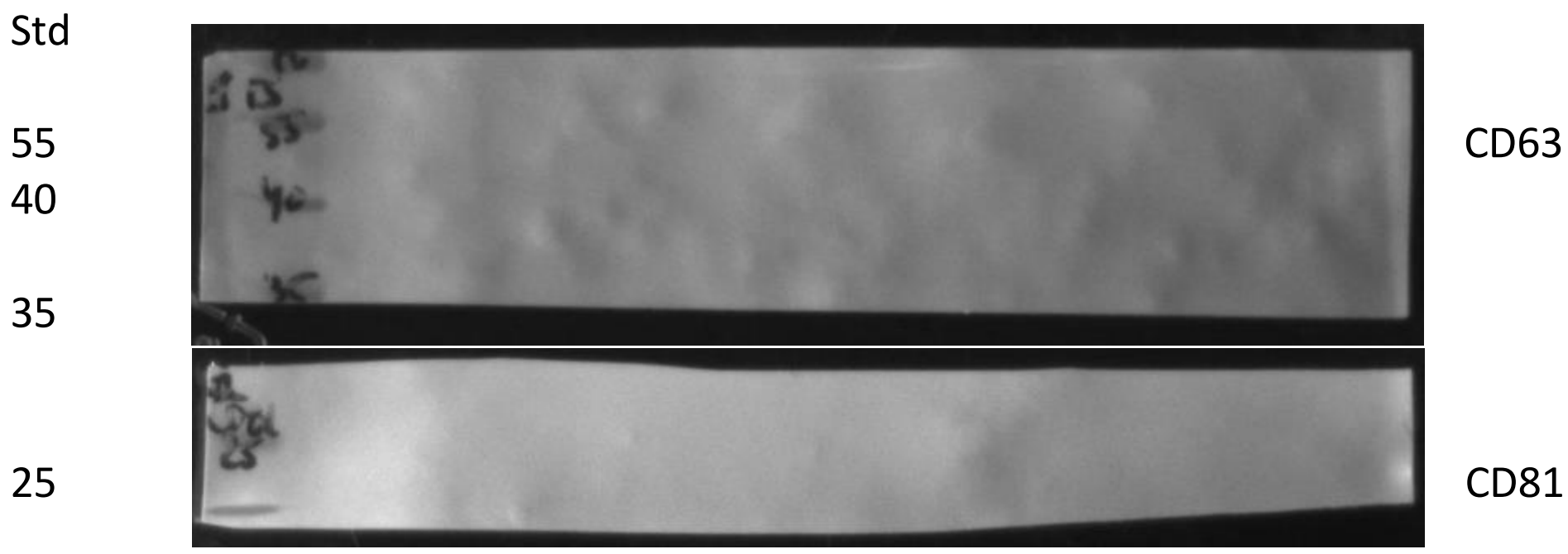

Figure 4F (Original immunoblot). N1 and N2 correspond to two independent isolations. N1 was used for the final image. Numbers 1 and 2 correspond to fractions 2 and 3, respectively, concentrated using filters treated with PVP-40, while numbers 3 and 4 correspond to fractions 2 and 3, respectively, concentrated using filters not treated with PVP-40. Sample X was lost during gel loading. The part enclosed in the red box was used for the final image

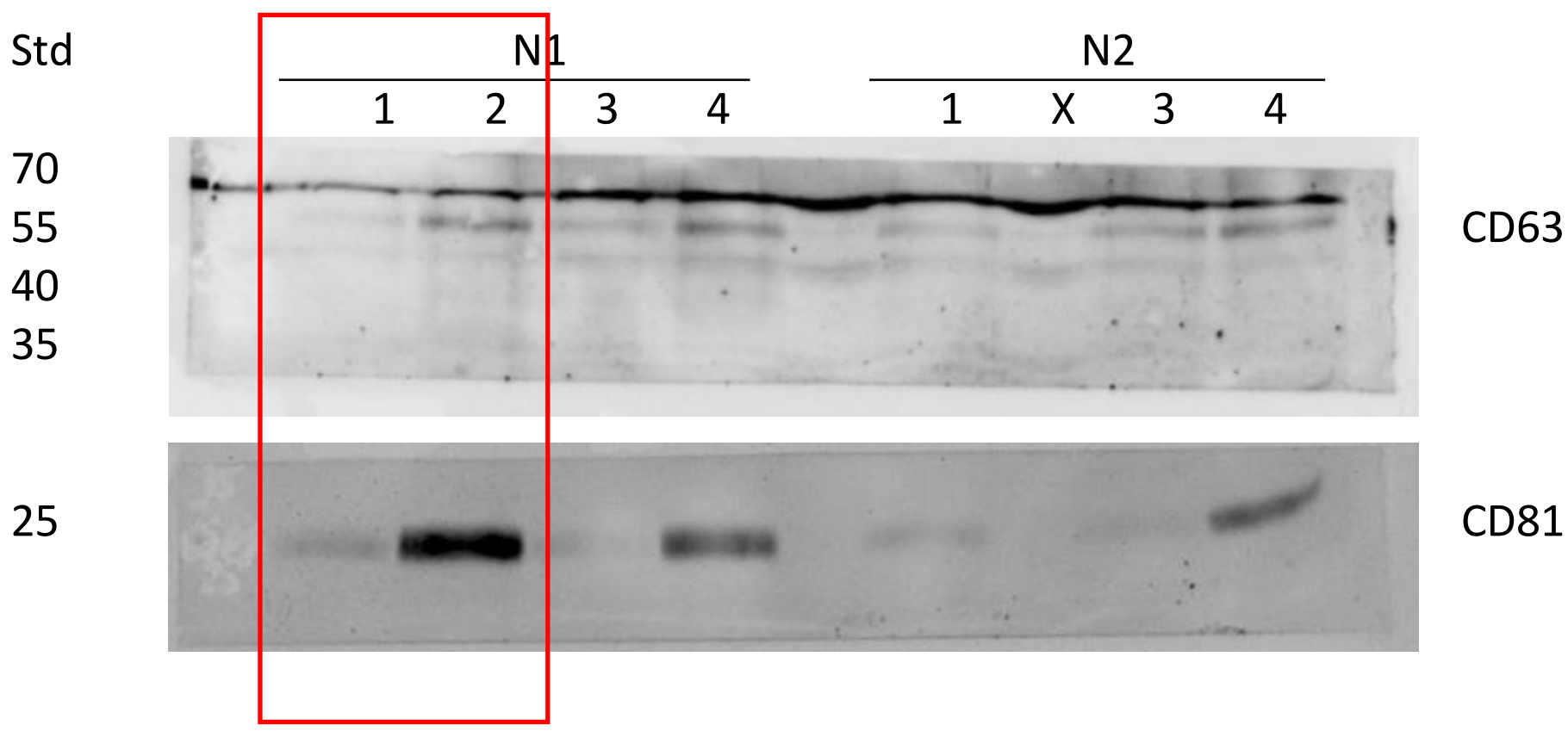

Photograph of the membrane, in which the molecular weight standard can be observed

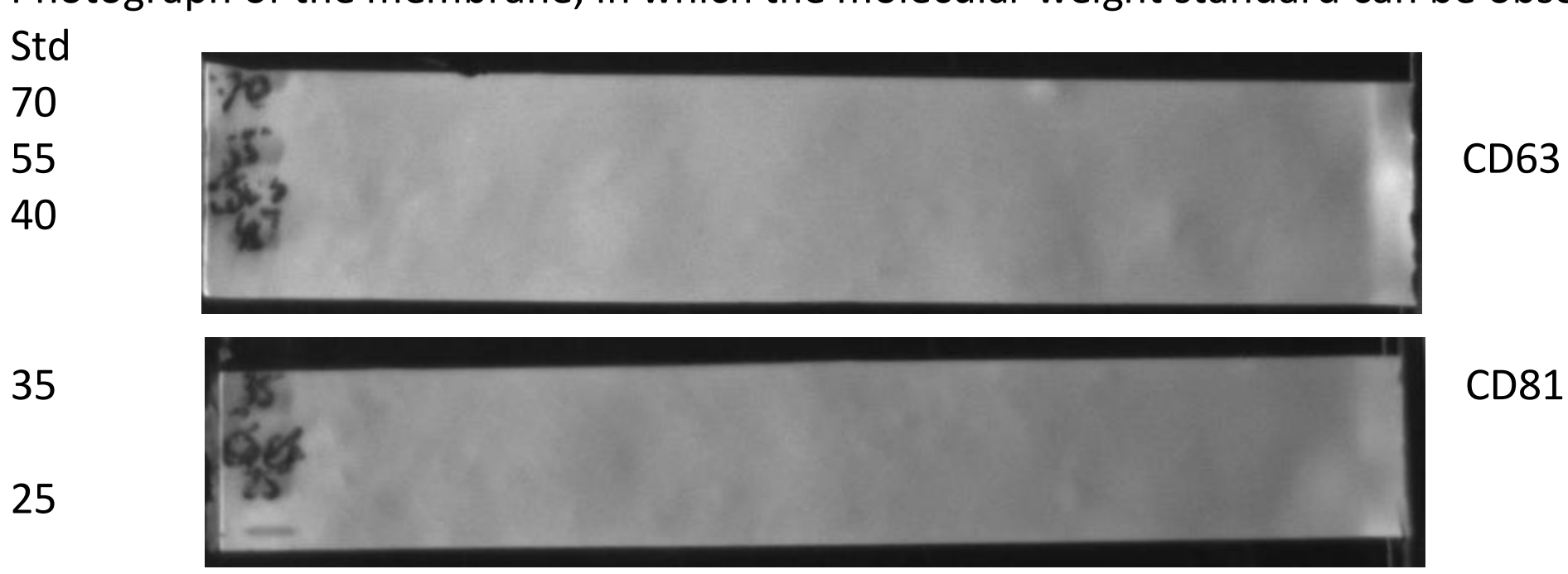

Figure 6A (Original immunoblot). The regions enclosed in the red box was used for the final image. The top part of the image corresponds to the immunoblot on this page, and the bottom image corresponds to the immunoblot on the next page.

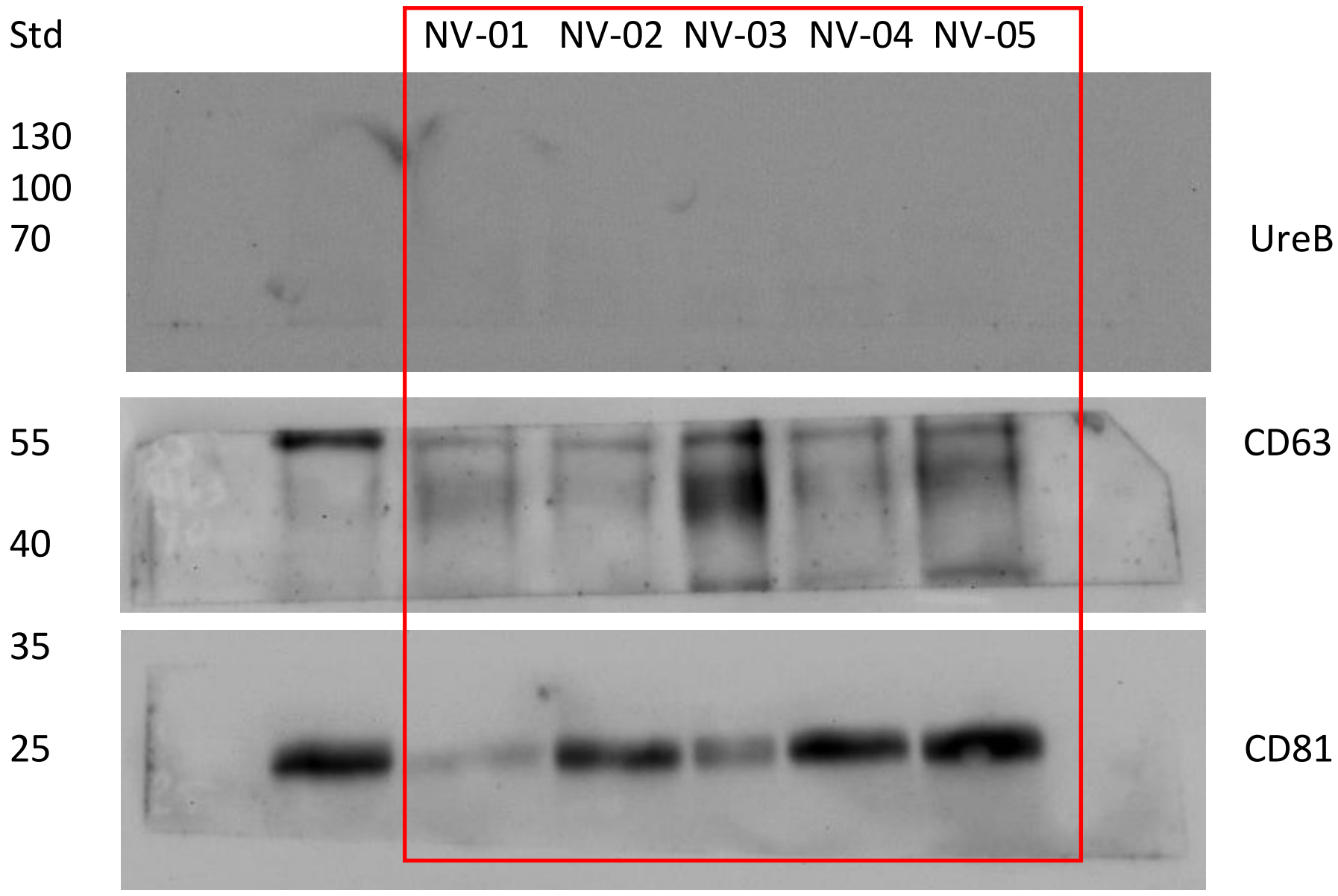

Photograph of the membrane, in which the molecular weight standard can be observed

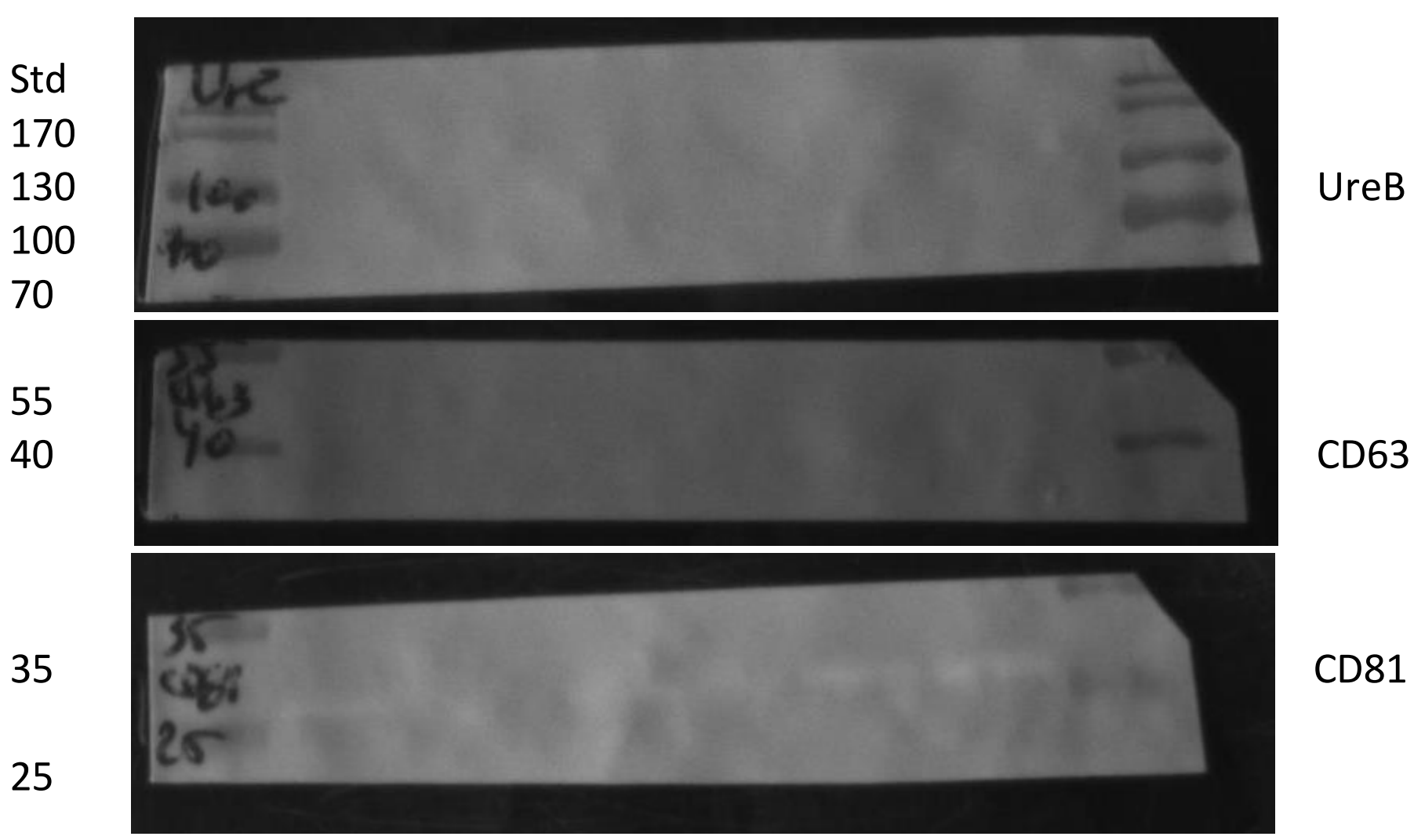

Std NV-14 NV-16 NV-17 NV-18 NV-19

170
130
100
70

55
40

35

25

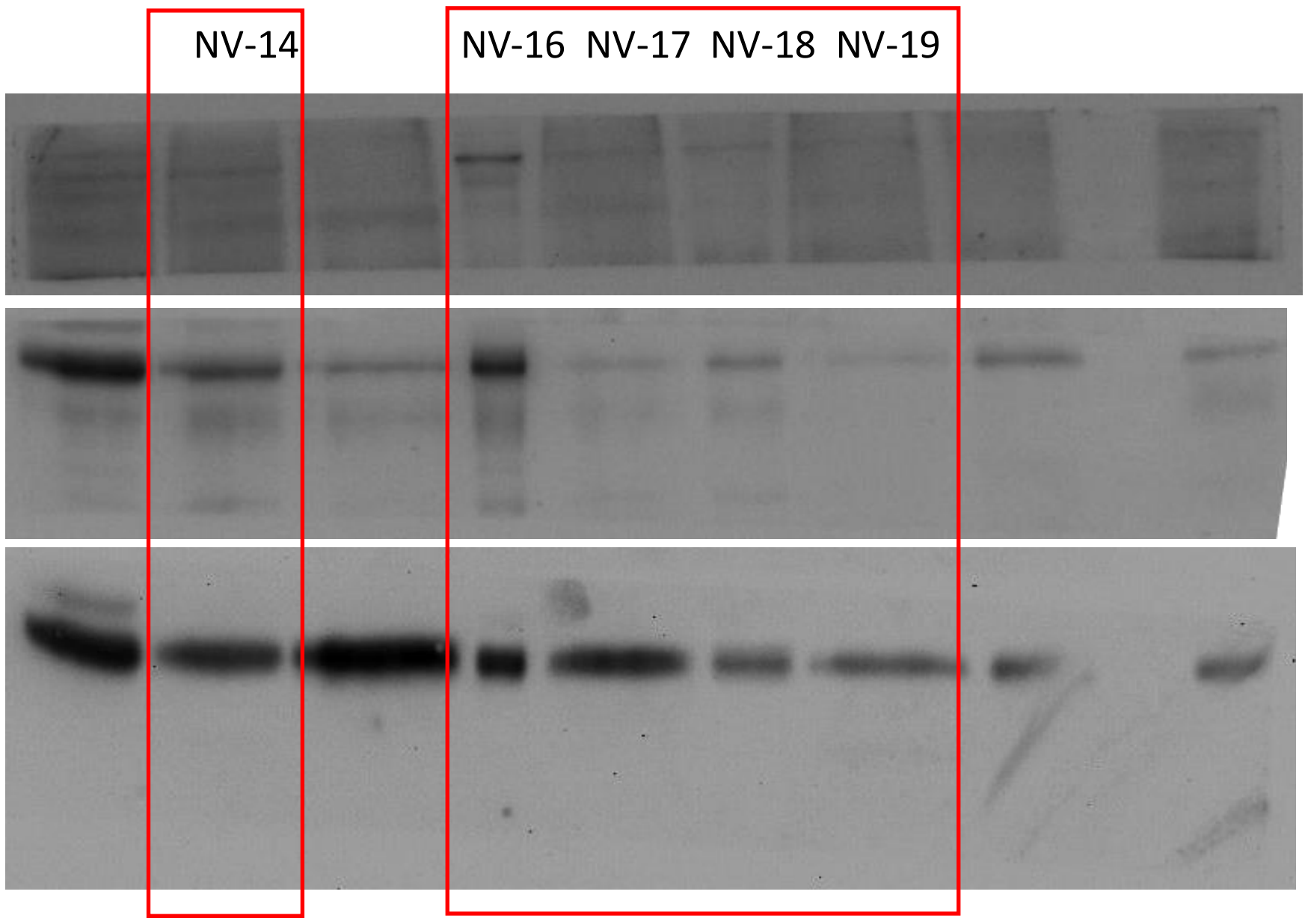

UreB

CD63

CD81

Std Photograph of the membrane, in which the molecular weight standard can be observed

170
130
100
70

55
40

35

25

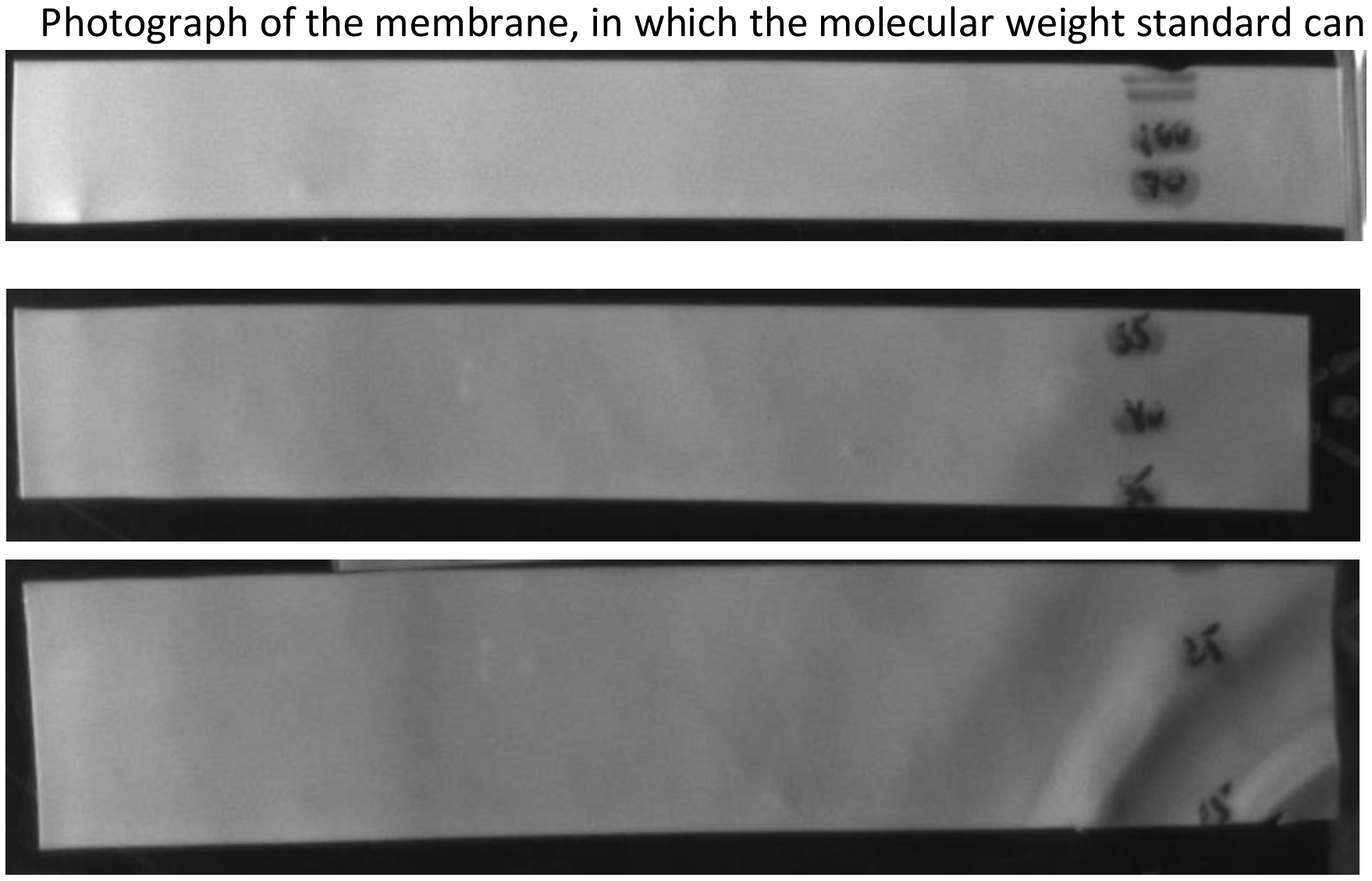

UreB

CD63

CD81
